# *Operando* Scanning Small-/Wide-Angle X-ray Scattering for Polymer Electrolyte Fuel Cells: Investigation of Catalyst Layer Saturation and Membrane Hydration– Capabilities and Challenges

**DOI:** 10.1021/acsami.3c11173

**Published:** 2024-05-13

**Authors:** Kinanti Aliyah, Christian Appel, Timon Lazaridis, Christian Prehal, Martin Ammann, Linfeng Xu, Manuel Guizar-Sicairos, Lorenz Gubler, Felix N. Büchi, Jens Eller

**Affiliations:** † Electrochemistry Laboratory, Paul Scherrer Institut, CH-5232, Villigen, Switzerland; ‡ Photon Science Division, Swiss Light Source, Paul Scherrer Institut, CH-5232, Villigen, Switzerland; § Institute of Physics, École Polytechnique Fédérale de Lausanne (EPFL), Lausanne 1015, Switzerland; ∥ Chair of Technical Electrochemistry, Department of Chemistry and Catalysis Research Center, Technical University of Munich, Munich 80333, Germany; ⊥ Department of Information Technology and Electrical Engineering, ETH Zurich, CH-8092 Zurich, Switzerland

**Keywords:** polymer electrolyte fuel cell, water management, catalyst layer, membrane hydration, operando small-angle X-ray scattering

## Abstract

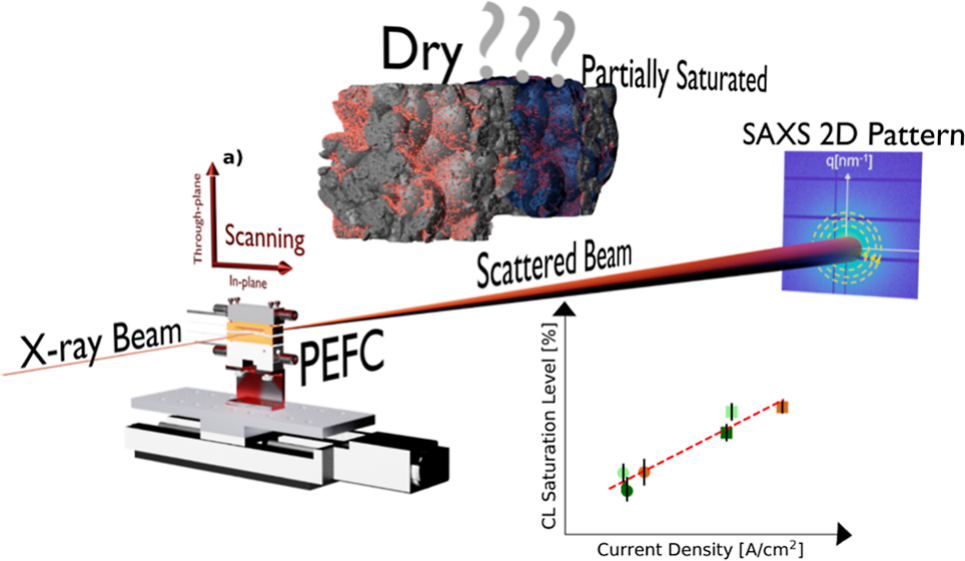

Polymer electrolyte fuel cells are an essential technology for future local emission-free mobility. One of the critical challenges for thriving commercialization is water management in the cells. We propose small- and wide-angle X-ray scattering as a suitable diagnostic tool to quantify the liquid saturation in the catalyst layer and determine the hydration of the ion-conducting membrane in real operating conditions. The challenges that may occur in *operando* data collection are described in detail—separation of the anode and cathode, cell alignment to the beam, X-ray radiation damage, and the possibility of membrane swelling. A synergistic development of experimental setup, data acquisition, and data interpretation circumvents the major challenges and leads to practical and reliable insights.

## Introduction

In recent years, considerable research and development efforts have been dedicated to enhancing the performance of polymer electrolyte fuel cells (PEFCs) as a favorable candidate for transport applications.^1^ Nevertheless, the power density still needs improvement for commercial success, with one of the core problems being water management.^2^ The intricate water balance in the PEFCs demands proper water management to guarantee efficient cell operation. Adequate membrane humidification is needed for high proton conductivity^3^ whereas excess water retention in the catalyst layer (CL) pores results in blockage of the gas diffusion to the active sites of the electrochemical reaction occurring in the CL, thus lowering performance and efficiency.^4^

Due to the small pores of the CL (2–200 nm, depending on the carbon support type),^5−7^ an accurate description of the water distribution in the CL remains challenging for direct neutron and X-ray imaging. However, with small- and wide- angle X-ray scattering (S/WAXS), the morphology of the solid materials and porous structures (pore size distribution, chord length distribution, and tortuosity) within this length scale can be probed and characterized thanks to the electron density difference of the components in the samples.^8−12^ So far, SAXS has been used in PEFC studies mainly for Pt-nanoparticle size changes^13−18^ and membrane hydration state determination.^19−22^ Furthermore, ultra SAXS was employed to determine the catalyst particle agglomeration in CL inks.^23,24^ Small-angle neutron scattering (SANS) has been used to study water saturation in the CL *in situ*.^25^ Recently, a high-energy X-ray *operando* cell, which is X-ray transparent in all rotating angles, has been developed to simultaneously image different components of the PEFC device.^26−28^ Using Compton scattering imaging with hard X-rays (100 keV or higher), the water distribution in GDL has been observed with suppressed radiation damage and sensitivity to lighter elements.^29^

**Figure 1:**
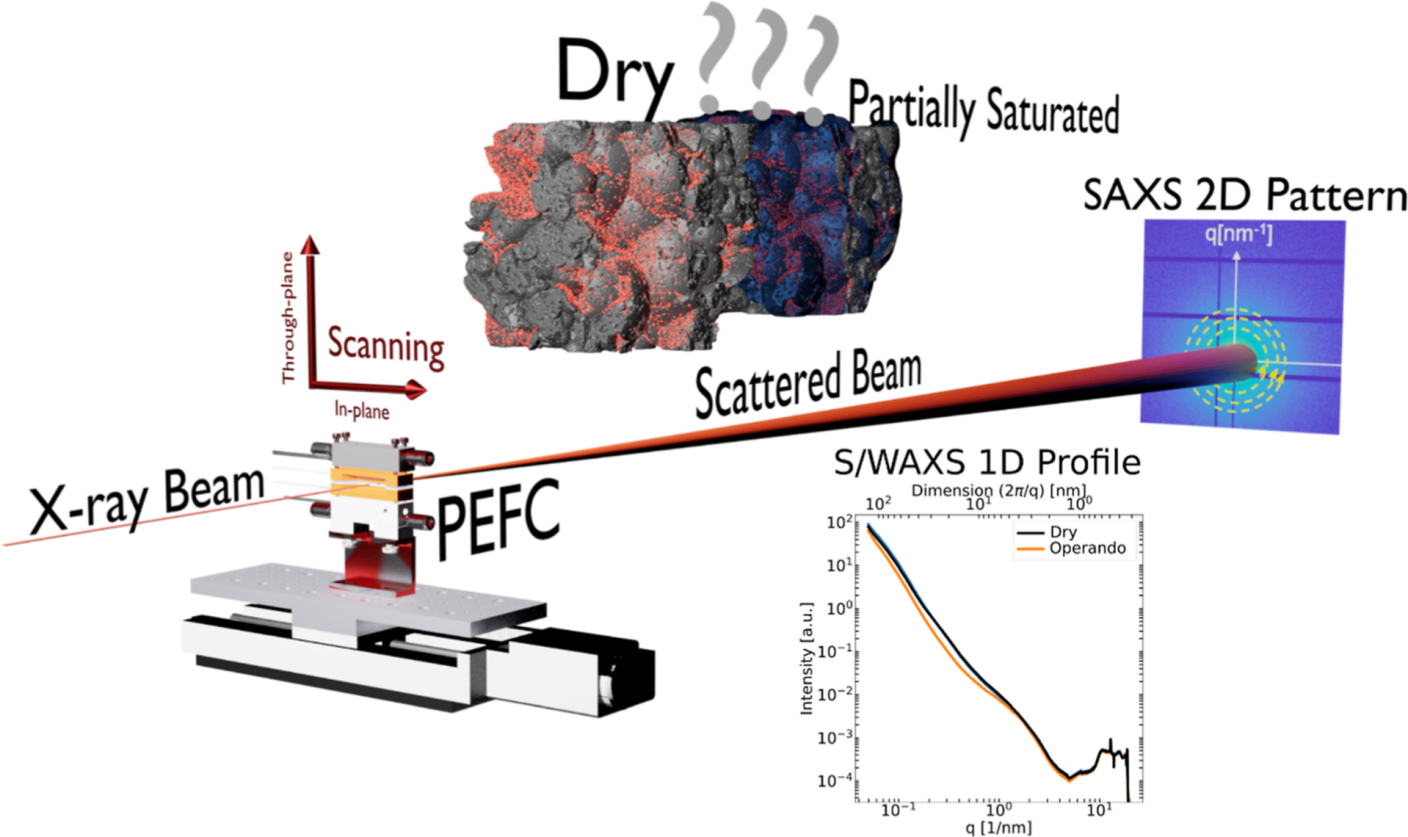
Schematic of the *operando* scanning SAXS PEFC approach. The porous PEFC components (CL, MPL) and the membrane are probed by a focused X-ray beam during operation. Based on the electron density contrast, the saturation level for CL/MPL or hydration state for the membrane can be revealed through the azimuthal integration of the SAXS 2D pattern/2D detector image.

In this study, we present a PEFC cell design for *operando* scanning S/WAXS^30,31^ in transmission mode that can provide pore size specific saturation and membrane hydration insights as schematically presented in Figure 1. The cell design and materials selection for the cell components, as well as cell alignment considerations, will be outlined. The sensitivity of the SAXS measurements to X-ray irradiation will be analyzed in terms of structural integrity and cell performance. Strategies to deconvolute the signal stemming from liquid water in the porous layers from misalignment due to membrane swelling in the SAXS data will be presented. These efforts culminate in *operando* scattering insights into the cathode CL pore size specific saturation levels as well as into the state of membrane hydration.

## Materials and Methods

### Operando Cell Design and Components

During scanning small-angle scattering measurements of the *operando* SAXS cell in the transmission mode, the cell is moved with respect to the beam in vertical and/or horizontal directions to measure separately the individual components of the membrane electrode assembly (MEA). Significant cell development efforts must be accomplished fulfilling the requirements of both fuel cell operation and interaction of the beam with the cell. For a reliable fuel cell operation, optimizing the electrical conductivity, mechanical stability, thermal management, and compression is crucial as pointed out by Kulkarni *et al.*(32) To obtain a good signal-to-noise ratio, the cell holder must be X-ray transparent as much as possible at the relevant X-ray energy. Therefore, the dimension of the MEA in the beam direction is limited by the X-ray transmission.

To ensure that the recorded signals originate exclusively from the materials of interest, the membrane and electrode’s precise alignment to the beam is indispensable for S/WAXS in the transmission mode. Therefore, additional geometrical measures may be employed to aid in the aligning process. For example, Lee *et al.*(25) used a pair of Cd wires with high neutron attenuation, inserted in upstream and downstream positions of the neutron beam for *operando* fuel cell SANS. The alignment was then verified by measuring the intensity of the beam transmitted. After the alignment, the pair of Cd wires was removed to avoid parasitic scattering from the wires. In retrospect, the requirement of repeated placement and removal of Cd wires near the active area may introduce unwanted complications during fuel cell operation.

Even when the cell is aligned properly, an additional problem could arise from the materials in the MEA. For example, with a wide channel in the flow field, the MEA can intrude into the channel. Additionally, the membrane may swell in the presence of water, complicating the data evaluation when comparing dry and wet states. With the recent technical developments going toward thinner membranes for high performance by reducing the ohmic losses while maintaining low hydrogen crossover and high mechanical stability,^33^ the challenge of separating the anode and cathode emerges. The spatial resolution of the scanning SAXS experiment then becomes a key factor that defines whether the cathode, membrane, and anode can be separated. The thickness of the CL, which usually is ≈ 7–20 μm, determines the upper limit of the beam height to probe only the isolated CL.

**Figure 2:**
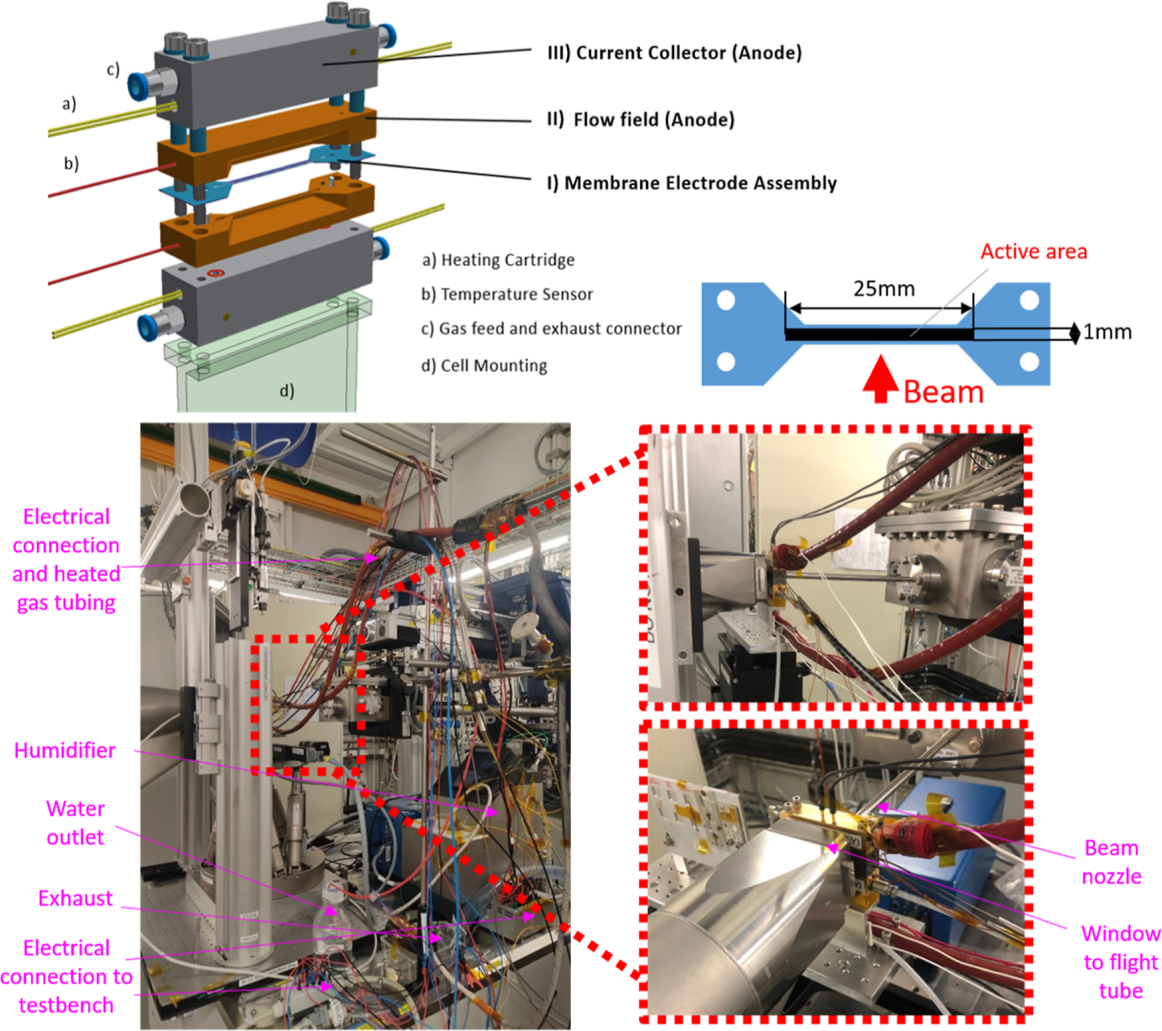
Top left: schematic design of the cell and its components. Top right: schematic design of MEA in the top view showing the active area dimension with respect to the beam. Bottom left: periphery of the *operando* cell at the beamline with the supporting devices labeled. Bottom right: close-up view of the cell with respect to the beam nozzle and flight tube.

The SAXS cell is composed of 3 main components—(I) MEA, (II) flow fields, and (III) current collectors (see Figure 2). The active area of the cell is defined by the dimensions of the CL, which are 25 mm (perpendicular to the beam) × 1 mm (parallel to the beam). The 25 mm long MEA perpendicular to the beam is required since X-ray exposure with a focused synchrotron beam may damage the probed area very quickly.^34−36^ The wide active area allows the use of pristine regions (multiple subsequent measurements by lateral shifting of the cell, hence always unexposed regions) for different operation conditions to avoid probing locations being degraded by previous X-ray exposure. The 1 mm width is chosen to retain good transmission and an S/WAXS signal-to-noise ratio through the platinum-containing CLs at a 11.2 keV beam energy which is below Pt absorption edges.

A laser-cut Kapton film is selected as a hard stop gasket for the MEA on the cathode and anode because of its X-ray transparency and the minor scattering contribution. The thickness of the gasket as well as the flow field shape also defines the compression of the MEA, depending on the GDL thickness employed. For the experiments in this study, 75 μm thick Kapton gaskets were used.

The flow field plates (Sigracet BMA5, SGL Carbon, Germany) on the anode and cathode are symmetrical and machined to have a 0.30 mm wide single channel and two 0.35 mm wide ribs. Additionally, two small holes near the active area are drilled into the flow field, such that alignment pins (indicated as white cylindrical objects in Figure 2) can be employed to assist assembly and alignment of the MEA to the flow field. O-rings are used for gas sealing toward the current collectors. Finally, a groove with a 100 μm depth is machined to the flow field where the GDL (25 mm × 1 mm) is located.

Gold-plated stainless-steel blocks act as the current collector plate, providing homogeneous compression to the MEA and forming the interface for gas inlets, exhausts, current/voltage, and temperature control devices. The gold plating protects the metal from corrosion and ensures good electrical contact to the BMA5 graphite flow field. Compression is provided by four torque wrench-adjusted bolts placed within hollow PEEK cylinders for electrical insulation between the anode and cathode. Essential for the *operando* SAXS measurements is the separation of the anode CL from the cathode CL. From the geometrical consideration, 30 μm (membrane thickness)/1 mm (active area width) = 1.7 degrees of alignment precision is at least needed. Therefore, it is required to have precise control of the alignment of the cell with respect to the beam. Two rectangular slits with defined dimensions are machined into the current collector at the interface of the bottom flow field and current collector. This allows transmission profiles across the slits to monitor the MEA alignment (see Figure S1). A hexapod with 6° of freedom (model M850, Physik Instrumente) is used for a precise rocking and rotating motion to control the alignment (see Figure S2).

The cell is equipped with heating cartridges placed in both current collectors and temperature sensors located in the flow fields that allow for temperature control of the cell, the anode, and the cathode. In addition, Festo quick connect fittings for gas-tight connection to 4 mm tubings are incorporated. The cell is mounted to the hexapod of the cSAXS beamline *via* U-shaped aluminum parts and an insulator made of plastic in between. The electrical connection to the cell is provided by two 2 mm plugs on each cathode and anode.

Both reactant gases (H_2_, purity 5.0 and synthetic air, purity 5.6 used in this study) fed to the anode and cathode are humidified to the target relative humidity (RH) by two Nafion tubing-based humidifiers and a thermostat (Haake Phoenix P1, Thermo Fisher Scientific, Germany). The gas piping from the humidifier to the cell is heated to maintain the dew point while gas outlets are not. Pictures of the cell mounted and operated at the beamline are presented in Figure 2, bottom.

All electrochemistry work at the beamline as well as pretesting in the laboratory was carried out with a custom-made mobile testbench. The testbench is equipped with a mass controller system for gas regulations, an electronic load for cell potential control, a Tsuruga AC-resistance meter, and a water bath thermostat for reactant gas humidification as previously mentioned. The integrated hardware was all interfaced with LabVIEW-based control software. The cells were operated in a constant voltage mode. For beginning of life and end of life polarization curves as well as the electrochemistry data during the scanning SAXS measurements, due to the constant voltage operation mode with an electronic load instead of a potentiostat, the high-frequency resistance was eventually less precise then during the constant current operation mode.

### Membrane Electrode Assembly

**Figure 3:**
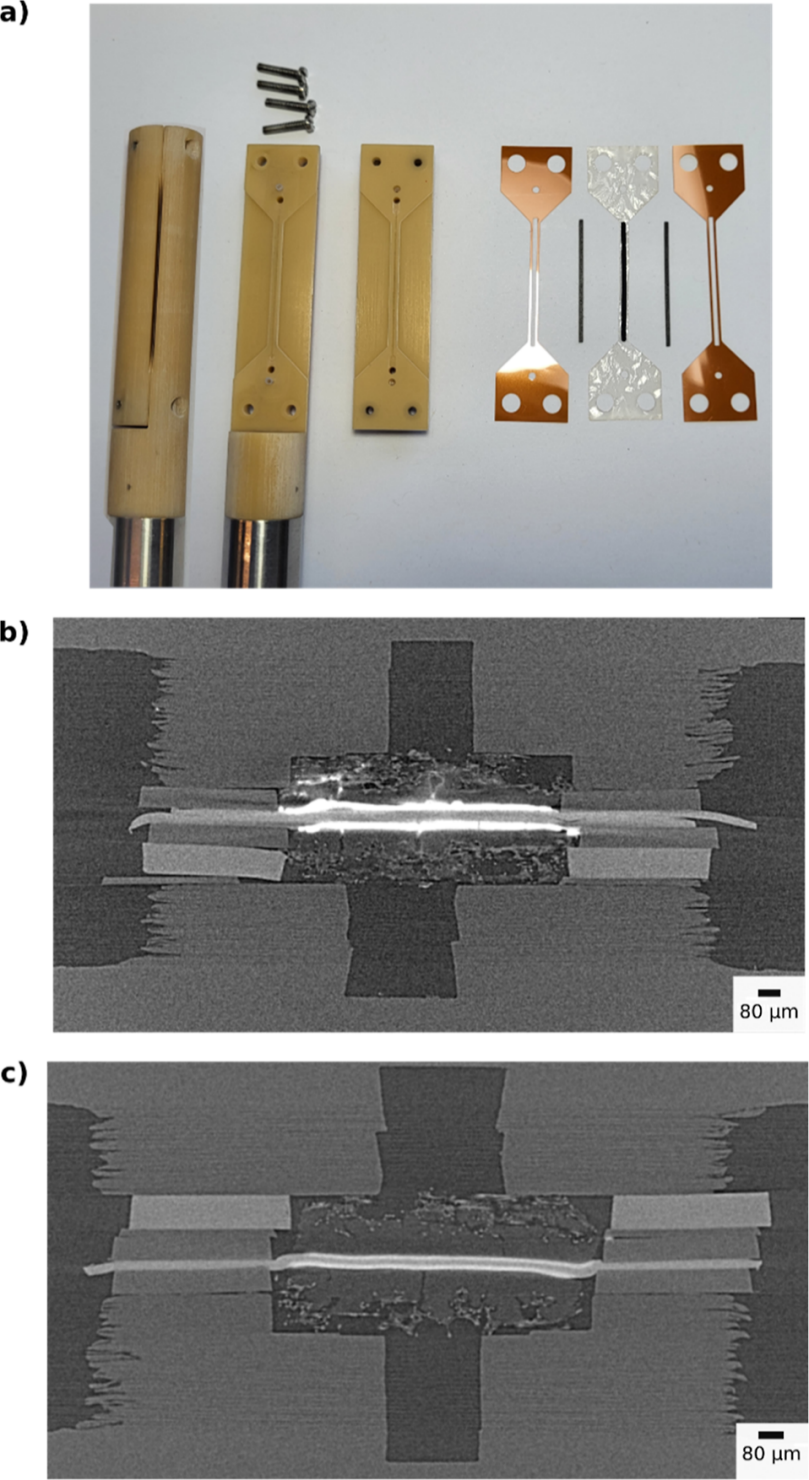
(a) X-ray tomography-compatible replica of the SAXS cell flow fields and MEA components before hot-pressing: Kapton gaskets, GDL stripes, and CLs already transferred on to the membrane (transparent). X-ray tomography of SAXS MEAs captured within the replica flow field for (b) two stacked Nafion XL membranes and high-loading CL and (c) one Nafion XL membrane and low-loading CL.

A 27.5 μm thick (according to datasheet) ePTFE-reinforced membrane (Nafion XL, Chemours) is employed to minimize membrane swelling related to data analysis issues. Thinner membranes were not considered for this study to ensure the clear separation of the anode and cathode CLs in the scanning SAXS data at this step size. Two different catalyst loadings of 0.1 mg_Pt_/cm^2^ (low) and 0.3 mg_Pt_/cm^2^ (high, Johnson Matthey gas diffusion electrode ELE0244-0542) were employed (symmetric loadings at both cathode and anode). For the high-loading MEA, two Nafion XL membranes were stacked while for the low-loading MEA, a single Nafion XL membrane was employed. The waviness of the MEA was sought to be minimized by using a narrow channel with 0.3 mm width only, achieving sufficient X-ray transmission calls for a narrow (1 mm) active area parallel to the beam. However, the small dimensions make the preparation and positioning of MEA components difficult. SGL 29BC (SGL Carbon, Germany) with a 235 μm initial thickness and 25% target compression was used as anode/cathode GDL. The low-loaded CLs were prepared by mixing catalyst powders (Pt/Ketjenblack, pore size distribution of the catalyst powder derived from nitrogen physisorption is reported in^6^) with 1-isopropanol and ionomer dispersion. Subsequently, the resulting slurry was coated onto PTFE decals. Scanning electron microscopy (SEM) images of the employed CL decal can be seen in Figure S3. The dried CLs (≈7–10 μm thick)^6^ were transferred to both sides of the membrane by hot-pressing the membrane with the CL decals. With the help of a computed tomography (CT)-compatible dummy flow field as shown in Figure 3a, the resulting flat MEAs as used in the SAXS experiments are visualized in Figure 3b,c, which show X-ray tomography slices of the MEA, recorded *ex situ* after the SAXS measurements.

### S/WAXS Data Acquisition and Processing

SAXS measurements were conducted at the cSAXS beamline of the Swiss Light Source at the Paul Scherrer Institut. A beam smaller, or not larger, than the CL thickness in the membrane through-plane (TP) direction is needed to probe only the thin CL. The spot size on the sample, as measured by scanning a sharp edge, was ≈7 μm vertically and ≈28 μm horizontally full width at half maximum. An evacuated flight tube was placed between the sample and the detector to reduce scattering by air. An X-ray energy of 11.2 keV was selected using a Si(111) double crystal monochromator. The beam was focused on the sample by combining a horizontally focusing monochromator crystal and a vertically focusing mirror. The X-ray fluorescence reflected from the 1.5 mm beam stop, which is proportional to the direct beam intensity, was recorded using a scintillation detector (Oxford Danfysik CyberStar) and further used for transmission correction of the scattering signal. SAXS profiles were recorded with a Pilatus2M (Dectris) detector with a sample–detector distance of 2.1 m, covering the *q*-range of 0.049 < *q* < 5 nm^–1^. A WAXS detector (Pilatus 300k, Dectris) was mounted at 0.64 m and 17.7 deg below the flight tube behind a Kapton window, covering a *q*-range of 6.5 < *q* < 20 nm^–1^.

A scanning pattern of 10 lines with 42 vertical (membrane TP direction) measurement points each with a 5 μm vertical step size (low-loading CL) or 10 μm vertical step size (high-loading CL with two stacked Nafion XL membranes), respectively, was employed to capture the different layers of the MEA while probing the 1 mm wide MEA perpendicular to the gas flow direction (see sketch of the active area and beam orientation in Figure 2). A 36 μm horizontal (gas flow channel direction) step size was used to capture the layers’ inhomogeneity in the in-plane direction. Three exposures with 0.3 s exposure time each to gain statistics were taken continuously one after another without a beam shutter closing in between at each motor position.

For every MEA location where *operando* S/WAXS data at a cell temperature of 50 °C were collected, dry references (SAXS line scans operated with nitrogen feed gas with a RH of 50% at both cathode and anode, no current produced) and subsequently wet references (SAXS line scans operated with nitrogen feed gas with a RH of 100% at both cathode and anode, no current produced) were acquired as well to enable the quantification of saturation and hydration during cell operation. The dry references particularly are crucial element to the study. Without the dry references, the water in the porous layers will not be quantifiable. Since collecting the reference condition S/WAXS data takes a considerable amount of the beam time (≈2 h per cell per condition), the scanned locations were instead scanned twice under different *operando* conditions. This allowed us to save time of another 2 h for the reference scanning of pristine *operando* spots and deemed necessary to complete planned measurements during the allocated beam time. This approach was implemented for the same cell voltage at different RH conditions (*i.e.*, the measurement spots for RH 100% 650 mV were at the exact place where RH 50% 650 mV data were first recorded). In summary, a 10-line scan location was scanned 4 times, at one operating voltage H_2_/air RH 50%, another operating voltage H_2_/air RH 100% followed by dry reference N_2_/N_2_ RH 50%, and wet reference N_2_/N_2_ RH 100%.

The 2D SAXS data containing scattering from the samples and Kapton gaskets were measured. The 2D SAXS data reduction was performed with cSAXS processing scripts to obtain azimuthally integrated 1D SAXS profiles.^37^ Subsequently, the raw 1D profiles of the samples were divided by the transmission counts. Then, the background signal measured from empty Kapton gaskets (no MEA in the cell), also divided by transmission, was subtracted from the raw scattering to obtain the pure scattering of the sample, except for the membrane area as there is no Kapton gasket on the beam path where the membrane is.

### S/WAXS Data Analysis

The CL saturation level was calculated by a three-phase invariant calculation using our Python code fcsaxs.^38^ The invariant calculation was only applied to data falling between *q*-range 0.05 < *q* < 5 nm^–1^, after the Pt contribution, which was regarded as constant, was subtracted from the data as described in.^39^ The Pt contribution was derived by fitting the Pt bump in the dry and wet data with log-normal distributed polydisperse spheres.

The porosity for invariant calculation is calculated by assuming the packing density of the carbon to be 28 μm/(mg_C_/cm^2^_geo_).^40^ In other words, a loading of 1 mg carbon per cm^2^ of the electrode area will result in 28 μm dry catalyst thickness.^40^ The ionomer-to-carbon mass ratio, set by the catalyst ink composition is 0.65, and Pt loading from the catalyst powder used is 0.2.^6^ Densities of carbon (skeletal), Pt, and ionomer used to calculate the volume fraction are 2, 21.45, and 2 g/cm^3^, respectively. Working out the mass ratio with the components’ densities considering the carbon packing density yields the volume fraction of carbon, Pt, and ionomer to be 17.9, 0.4, and 11.6%, respectively. The scattering length density at 11.2 keV of carbon + ionomer solid phase (obtained from the volume-averaged ionomer and carbon scattering length densities) for the invariant calculation is 17.3 × 10^10^ cm^–2^.

The separation of hydrophilic and hydrophobic domains in the ionomer of the membrane judged by the *d*-spacing was calculated by fitting the ionomer peak region by Porod’s law (*I* ∼ *Aq*^–4^ + *C*, *A* is the slope and *C* is the background) and a Gaussian peak.^41^ The *D*-spacing value is the real-space location (2π/*q*) where the Gaussian peak has its maximum.

### X-ray Tomography Data Acquisition

X-ray CT scans of the MEAs that were used for the scanning S/WAXS measurements were performed using a Nanotom-m Lab-CT scanner (GE Sensing and Inspection Technologies GmbH) at PSI. The samples were imaged while placed in a CT-compatible dummy flow field by using an acceleration voltage of 80 kV and a current of 230 μA. For each scan, 1800 projections were recorded during the 360° sample rotation. Each projection consists of two averaged images with an exposure time of 0.5 s each. A 33-fold geometric magnification was used, which resulted in a final voxel size of 3 μm.

## Results and Discussion

In order to realize successful *operando* SAXS measurements, a few prerequisites have to be fulfilled, and some challenges have to be solved. First, the separation of the anode and cathode CLs should be verified based on the X-ray transmission data. The MEA components should be identified by their specific scattering features. Since the MEA and PEFC performances are sensitive to X-ray radiation, the influence of the irradiation should be elucidated. Next, the uncertainty in CL saturation due to membrane swelling and CL misalignment should be addressed. Finally, a high confidence in the elimination of any measurement bias is needed. All this allows the first SAXS-based quantification of PEFC cathode CL saturation values and membrane hydration levels at selected operating conditions.

### Separation of Anode and Cathode Catalyst Layers

**Figure 4:**
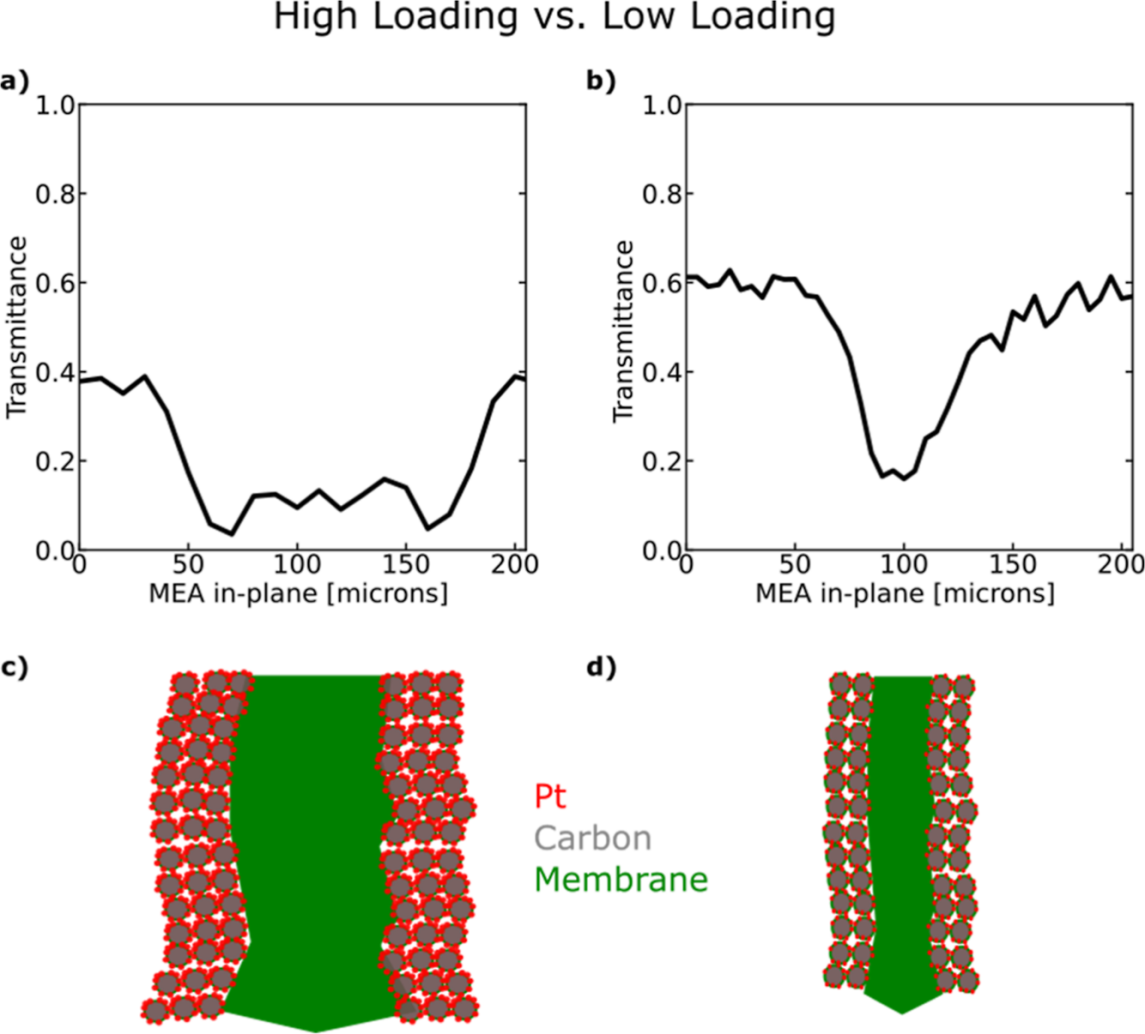
X-ray transmittance for two different MEAs: (a) two stacked Nafion XL membranes (total thickness of 60 μm) sandwiched by high-loading anode and cathode CLs (both 0.3 mg_Pt_/cm^2^) and a scanning step size of 10 μm. (b) One Nafion XL membrane (total thickness of 30 μm) sandwiched by low-loading anode and cathode CLs (both 0.1 mg_Pt_/cm^2^) and a scanning step size of 5 μm. Schematics of the CL and membrane location according to the X-ray transmittance: (c) high-loading CL and (d) low-loading CL. The lower transmission in the MPL of the high-loading CLs is likely due to catalyst depositions into MPL cracks (see Figure 3b).

As pointed out in the Materials and Methods section, with a cell that is poorly aligned with respect to the beam, anode and cathode separation may not be achieved. However, the transmission counts of the diode on the beam stop can be a good first indicator of the MEA alignment. For example, with a high-loaded CL (0.3 mg_Pt_/cm^2^), 2-fold stacked Nafion XL (≈30 μm × 2), and a scanning step size of 10 μm, the transmission values indicate a successful separation of anode and cathode CLs as the absorption from Pt in the CLs was considerably higher than the membrane and MPL (see Figure 4a). For the low-loaded CL (0.1 mg_Pt_/cm^2^), one layer of Nafion XL (≈30 μm), and a scanning step size of 5 μm, it is challenging to judge from the transmitted counts if the anode and cathode CLs were separated well (see Figure 4b). The transmittance from both the membrane and CL was at a similar level even though an arrangement as depicted in Figure 4c,d is likely. Hence, the SAXS data, in addition to the transmission signal, are needed to clarify the location of the MEA layers and the possibility to identify them. For this study, we focused on the low-loaded CL where higher transmittance provides more reliable scanning SAXS data.

**Figure 5:**
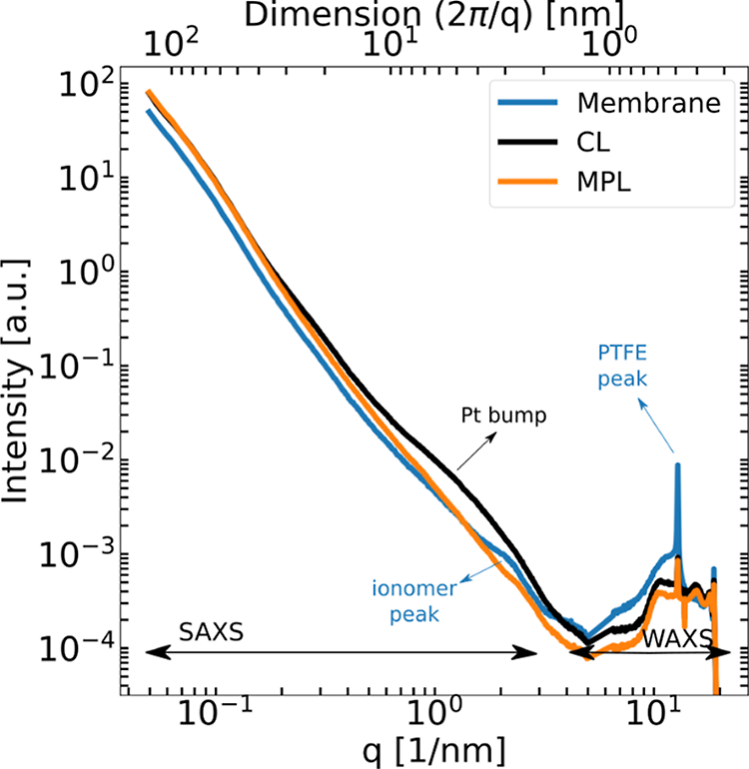
S/WAXS profiles of the different MEA components scanned in the *operando* cell.

The three fuel cell components scanned by the X-ray beam are the membrane, CL, and microporous layer. All components have different compositions and distinguishable features eminent in their corresponding SAXS profile even with a low Pt loading of 0.1 mg_Pt_/cm^2^ (see Figure 5). The membrane Nafion XL is a multilayer composite with outer layers of perfluorosulfonic acid (PFSA)-based polymer and with a PTFE-rich reinforcement in the middle.^19^ The membrane can be distinguished by the PTFE crystalline peak in the WAXS region (*q* ≈ 12.8 nm^–1^)^19^ stemming from the PTFE-rich reinforcement of the membrane and a PTFE amorphous shoulder (*q* ≈ 10 nm^–1^), as well as the ionomer peak corresponding to hydrophobic and hydrophilic domain correlation distance (*q* ≈ 2 nm^–1^).^19^ The CLs are based on porous carbon (30–100 nm particle size) with Pt nanoparticles and a PFSA-based ionomer binder. It can be recognized by the prominent Pt bump at *q* ≈ 1 nm^–1^. The microporous layer is composed of porous carbon with a similar particle size as the CL but without the Pt nanoparticles and with a PTFE binder instead of a PFSA-based binder. The MPL’s SAXS profile is following a power-law relationship, without the Pt bump.^39^

**Figure 6:**
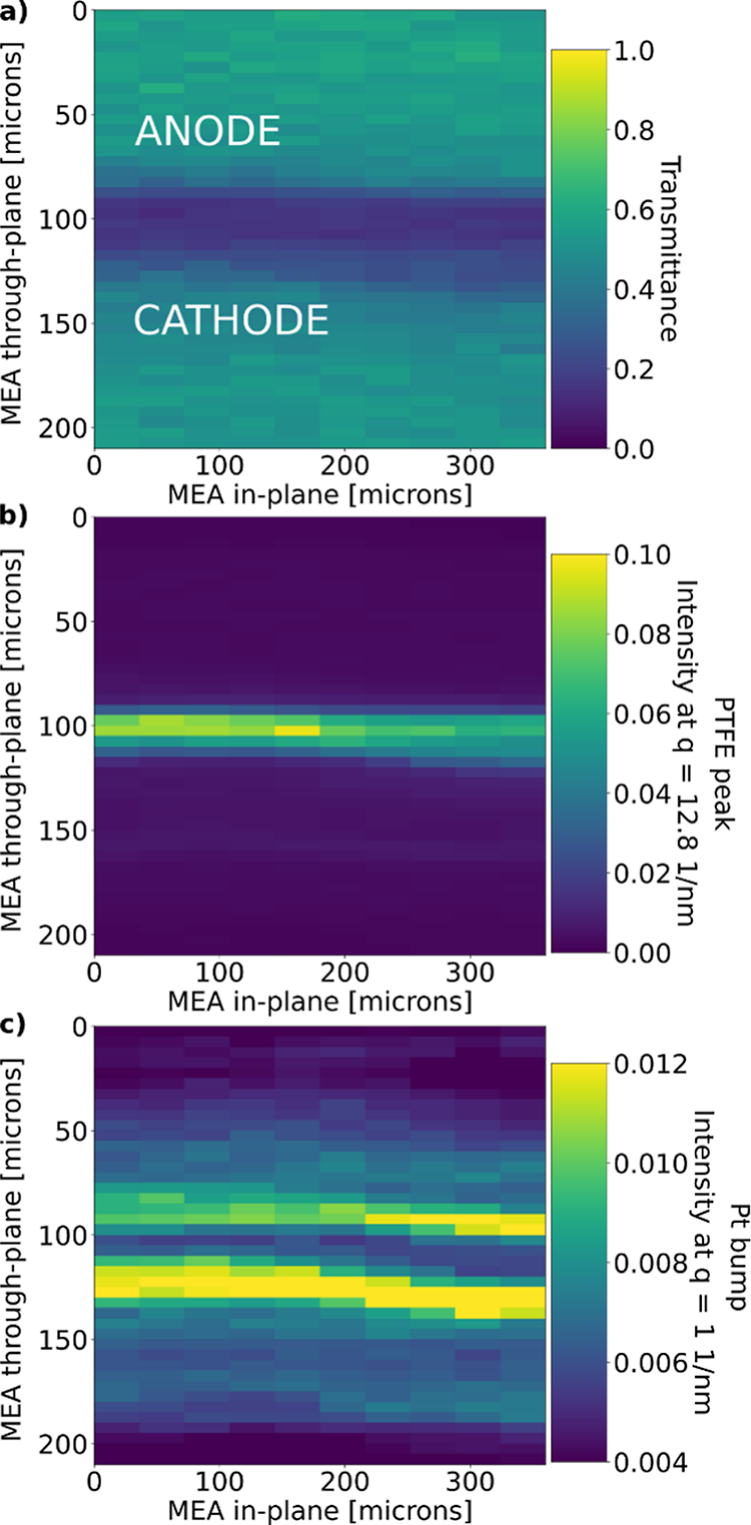
2D maps of (a) transmittance data allows us to roughly locate a MEA with the low-loading catalyst, (b) PTFE crystalline peak to locate the membrane’s reinforcement layer, and (c) Pt bump intensity to locate the CLs.

The different MEA layers can be identified accordingly as shown in Figure 5 by tracking the intensity counts at different *q* values corresponding to the most distinguishable features of the three components, namely, the presence/absence of the Pt bump and the PTFE crystalline peak. The transmission values can pinpoint the MEA’s position during preliminary alignment of the scan as the membrane and CL have higher absorbance than the MPL (see Figure 6a, also in Figure 4). However, it is nearly impossible to separate the membrane and CLs from X-ray transmission data for the low-loading CLs as mentioned earlier. From the intensity of the PTFE crystalline peak, the membrane can be precisely located by the significantly more intense PTFE peak signal from the membrane reinforcement layer than from anywhere else in the cell (Figure 6b). All the neighboring pixels of the membrane’s center of the membrane display the PTFE peak, albeit with much lower intensity (10x lower) compared to the PTFE peak in the center of the membrane and therefore display only dark colors of the color bar (see Figure 6b). The intensity profiles in Figure 5 are the representative profiles; all components in the study behave quite similarly to the ones in Figure 5.

However, there is still a peak attributed to PTFE in the CL domain that most likely stems from the membrane clamped between the gaskets in the beam path of CL (see Figure 3c). To estimate the contribution of the membrane in the beam path, an assumption of the volume fraction being 100% in the membrane is needed. If PTFE peak intensity of the membrane is assumed to be 100% membrane in volume fraction, and the pure CL to be 0%, the volume fraction of the membrane in the beam path can be estimated with the scaling factor between the PTFE peak in the CL and PTFE peak in the membrane as conducted in a previous study leveraging the WAXS peak for a quantification effort.^42^ In Figure 5, where the intensity profiles of the different components normalized by their transmission are depicted, the PTFE peak intensity of the CL is ≈ 0.01, whereas the PTFE peak of the membrane is ≈ 0.1. The membrane in the beam path is expected to be 2 mm, whereas the CL is expected to be 1 mm (see Figure 3). Hence, 1 mm of 100% volume fraction of a membrane would correspond to a PTFE peak of ≈0.05. The volume fraction of PTFE aligned with the CL domain can be estimated by 0.01/0.05 or to be about 200 μm, stemming most likely from the membrane clamped between the Kapton gaskets and minor misalignment between the active cell area and the membrane under the gaskets (see Figure 3c).

The Pt bump (intensity at *q* ≈ 1 nm^–1^) allows us to identify the CLs (Figure 6c), where a higher signal (marked yellow) coming from the Pt nanoparticles clearly separates them from the membrane and MPL area. The parallelism of the membrane and CLs to the beam is best in the cell, where the corresponding signals are strongest, at about the 250–350 μm in-plane MEA position. If the MEA orientation deviates from the beam direction, then the Pt signal appears blurred in the scanning SAXS data as is the case for lower in-plane MEA positions of 0–150 μm as shown in Figure 6c. Nevertheless, the alignment of the MEA to the beam was sufficient to separate the MEA components based on the allocation of the membrane (PTFE peak) and CLs (Pt bump) of the S/WAXS data for all scanned locations.

While their locations are consistent and agree well with the X-ray transmission data and give evidence for a successful layer separation, the intensities of Pt bump at the anode CL and cathode CL are different, despite the same CL loadings used (see Figure 6c). Such differences could stem from different reasons. For the different MEA in-plane locations, such differences in Pt bump intensity could be attributed to the differences in MEA-to-beam parallelism, which would effectively decrease the CL in the beam path and thereby the scattering intensity. At fixed MEA in-plane locations, the deviations of MEA-to-beam orientation would be only possible in the case of significant membrane thickness variations in the beam direction, which seems unlikely for beginning-of-life MEAs as used for this work. Differences in the effective amount of Pt in the beam path could result from local deviations in sample width (presumably less than 10%), lower CL thickness, or the Pt/C ratio of the catalyst powder (very unlikely). Considering the fluctuations of the Pt bump intensity for different scanning SAXS 2D mappings at the same cell location but different cell operation and humidification conditions (see Figures S4 and S5), it becomes evident that the raw Pt bump intensity cannot be used for the quantification of local Pt amounts. Eventually, an extensive analysis of the Pt bump fitting done during the CL saturation determination could allow such a quantification, but this was not implemented in this work.

The thickness of the CLs and the membrane in the TP direction can be analyzed for the locations with high MEA-to-beam parallelism expressing the high intensity of the Pt bump and PTFE peak for the CL and membrane, respectively. Both at the anode and cathode CLs, such locations mainly were 2–3 neighboring data points reflecting a strong Pt bump signal in the TP direction (see Figure 6c at about 250–350 μm in-plane MEA position). The vertical step size of 5 μm between the data points would translate to 10–15 μm CL thickness with an expected CL thickness of 7–10 μm based on SEM and micrometer thickness probe analysis. The deviation between the methods most likely stems from the Gaussian shape of the beam with a full width at half maximum of around 7 μm in the vertical direction and is within the range of what to expect of a Gaussian-blurred ground truth Pt signal. Some locations where the CLs appear to be just one pixel in the TP line scans could either represent a locally thinner CL or result from a perfect overlap of the scanning position and CL center.

Similarly, the thickness of the membrane can be examined from the PTFE peak (Figure 6b). There are 5–6 neighboring data points with high PTFE intensity, corresponding to a PTFE reinforcement thickness of 25–30 μm. The membrane TP thickness is expected to be 30 μm (Nafion XL). Accordingly, the Gaussian profile of the beam also leads to the impression of a thicker than expected reinforcement layer as the ionomer layers of the membrane are expected to generate a significantly less PTFE peak. When comparing the data points of the PTFE peak and Pt bump, they overlap, particularly at the anode CL part. The overlap may come from the aforementioned imperfect sharpness of the beam or more presence of the membrane clamped between the Kapton gasket on the beam path of the anode CL. The latter effect would also lead to the false suggestion that the cell area shown in Figure 6 has a highly parallel MEA-to-beam orientation at low in-plane MEA positions and imperfect MEA-to-beam alignment at higher in-plane MEA positions. However, this interpretation of the MEA-to-beam alignment would contradict the understanding of the Pt bump signal beforehand. The purely MEA-to-beam interpretation is incorrect here as one has to consider that the membrane can contribute from locations with different alignments to the beam, namely, the active area and the area where the membrane is clamped between the gaskets (see Figure 3c). At low in-plane MEA positions as shown in Figure 6, the membrane between the gaskets and the active areas is supposedly located at more or less the same TP position, which results in a sharp PTFE signal with high intensity. In contrast, the membrane between the gasket and the active area is offset by about 10 to 15 μm at high in-plane MEA positions, which results in a broader PTFE peak at a lower intensity and falsely suggests a nonideal MEA-to-beam alignment.

For this study, we limit the following SAXS data analysis to the determination of the cathode CL’s water saturation and the hydration level of the membrane.

### Assessment of Radiation Damage

**Figure 7:**
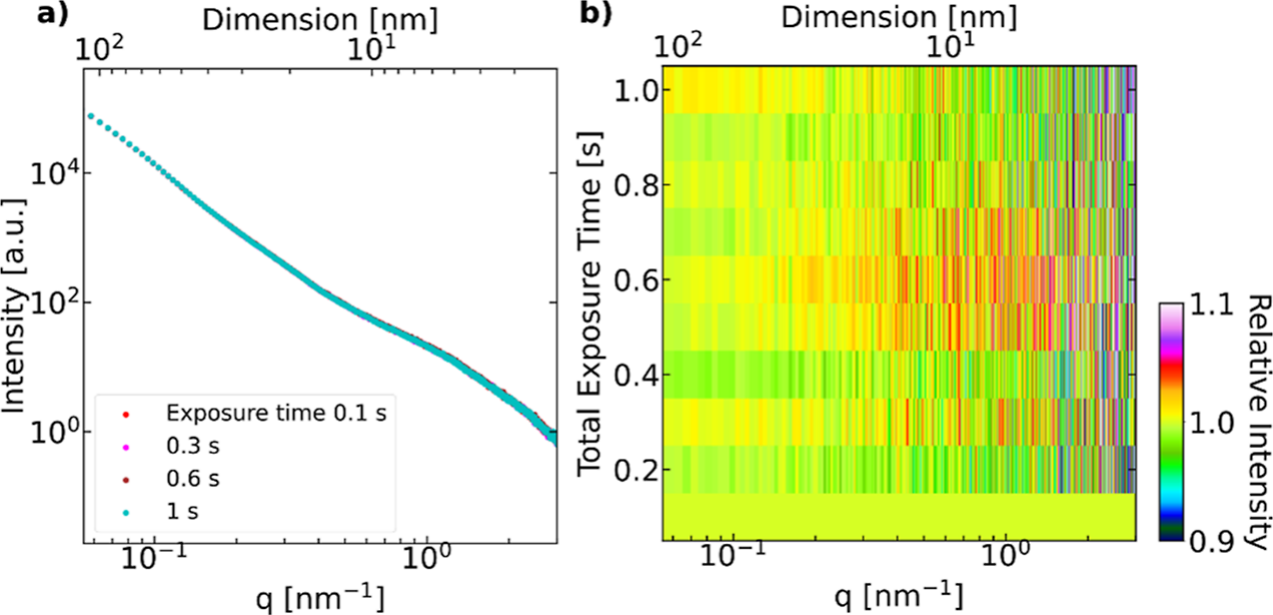
(a) Intensity profiles of the CL at different accumulated exposure. (b) Relative intensities of the CL over the exposure time (normalized by the 0.1 s total exposure time).

X-ray irradiation has been previously shown to induce chemical decomposition, especially in the membrane and the ionomer of the CL.^32,34,36^ The CL was irradiated 10x with 0.1 s exposure (1 s in total) to assess the presence of morphological changes due to X-ray irradiation. There were no significant changes to the SAXS profiles with increasing exposure time to 1 s (see Figure 7), and the noise follows Poisson noise distribution. Hence, the exposure time used for *operando* SAXS measurement <1 s can be considered harmless to the morphology of the CLs.

**Figure 8:**
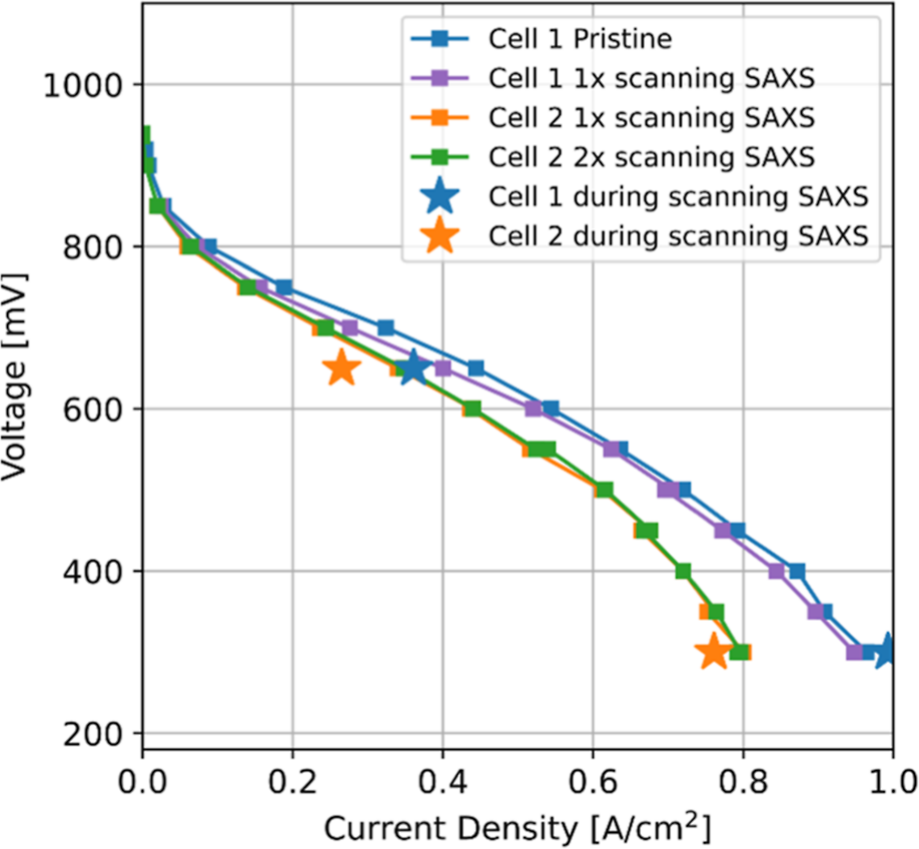
Polarization curve of a pristine cell (cell 1 pristine), after 1 set of scanning SAXS (Cell 1 1x scanning SAXS and cell 2 1x scanning SAXS) and after scanning at different locations (cell 2 2x scanning SAXS). The fuel cell was operated at 50 °C, ambient pressure at the outlet, and a 100% RH. All points in the polarization curves were taken in the constant voltage mode with 60 s hold. Current densities during scanning SAXS measurements are indicated by the star symbol.

The influence of X-ray irradiation on the PEFC performance is addressed by an analysis of polarization curves of pristine and irradiated MEAs (constant voltage mode with 60 s hold). The polarization curve taken after the SAXS measurements on 14% of the active cell area (including 2x operando scans and 1x dry and 1x wet reference scan of each scanned location) does not significantly differ from that of the pristine state (see cell 1 in Figure 8). By operating the cell at constant voltage, a possible bias on the local current density of a spot to be scanned by local performance losses of previously scanned cell domains can be omitted, since it is expected that the pristine area behaves according to the overall pristine cell polarization curve. Another MEA was scanned twice at different locations to understand the cell-to-cell and in-cell repeatability (cell 2 *vs* cell 1 in Figure 8). The in-cell repetition reveals similar performance with again negligible differences when running the same operation and scanning protocol once or twice. Detailed time series data from the operation of the two cells at the beamline can be found in Figures S6 and S7 (voltage, current density, high-frequency resistance, and RH versus time). The cells (cell 1 and cell 2) at 650 mV were not in the steady state even after 20 min equilibration. However, at 300 mV, 20 min equilibration time yielded quite stable current densities. Due to the limited beam time, there was unfortunately not enough time to further equilibrate the current densities at 650 mV.

However, the variability in performance between the two MEAs of the same composition is significant. This is most likely due to the fact that the MEAs were conditioned in the laboratory, one by one, in the only available SAXS-compatible cell. Thereafter, the MEAs were removed from the cell, stored in plastic zip-lock bags, and put back into the cell at the beamline for the SAXS measurements. The disassembling and reassembling of the cell were implemented because assembling fresh MEAs at the beamline, subsequently conditioning with the used several-hour-long protocol (>8 h) and reproducing good cell alignment to the beam (>1–2 h), was considered as too time-consuming. Unfortunately, the cell performance after disassembling and reassembling was always lower than after preconditioning which caused a wide variability between cell repetitions. The repeatability of the cell performance was much better immediately after preconditioning and before MEA removal from the cell (Figure S8) and acceptable considering the small active area. For future studies, short preconditioning protocols (≤1 h) that can be adopted at the beamline have to be implemented to improve cell performance and reproducibility.

### Membrane Hydration

The PEFC membrane hydration level has been extensively investigated because of the easily distinguishable and interpretable membrane ionomer diffraction peak.^19,26,41^ Hence, the investigation of the membrane hydration level is an excellent measure to confirm the capabilities of the newly developed *operando* cell.

**Figure 9:**
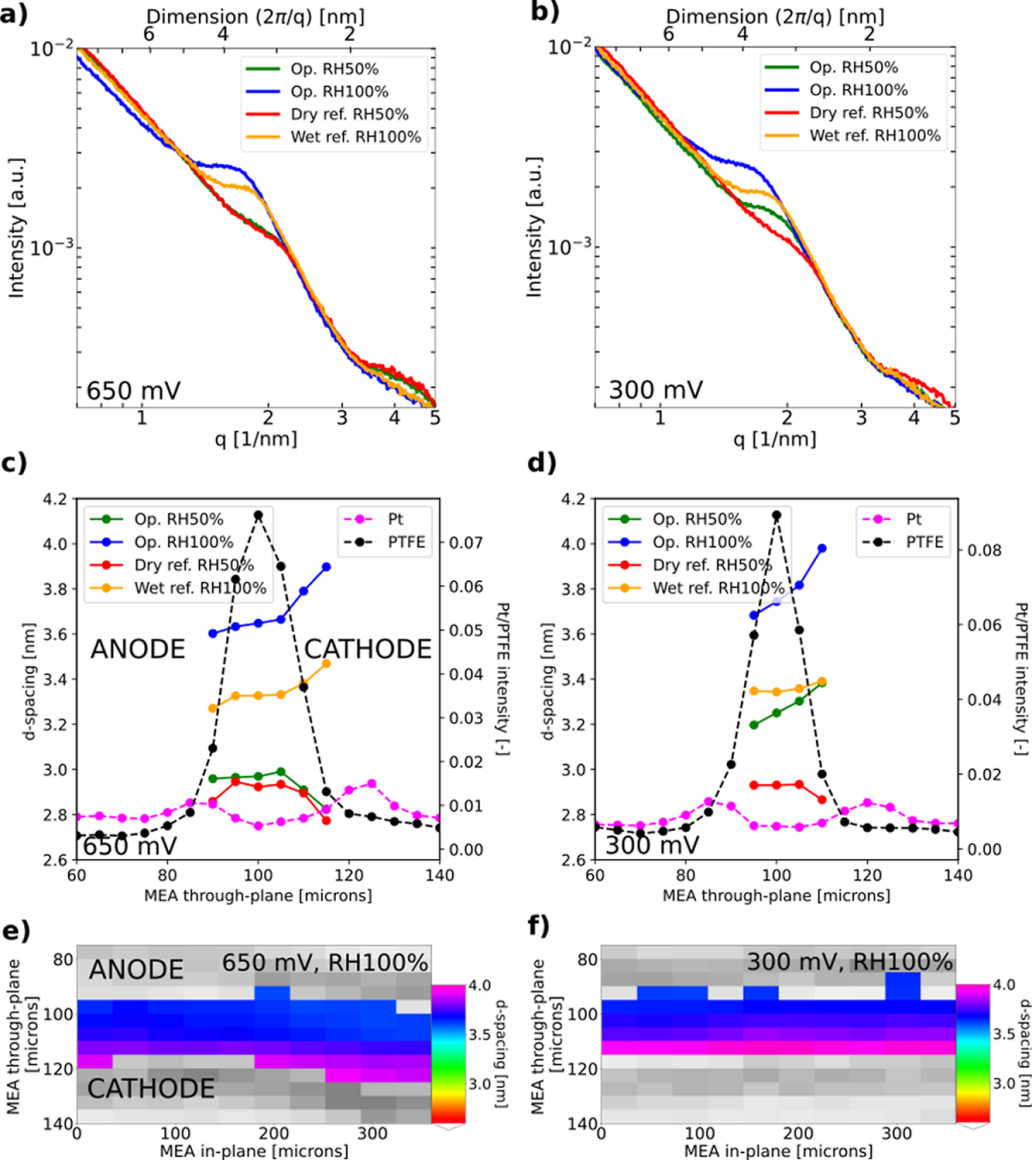
Top: representative SAXS profiles (taken from cell 2) of the membrane area at (a) 650 and (b) 300 mV condition/location, both at RH 50% and 100%. Middle: *d*-spacing line profiles of membrane TP direction from the anode (left) to the cathode (right) at (c) 650 and (d) 300 mV condition/location, scanned at different locations. Bottom: 2D maps of the *d*-spacing distribution of the scanned area for *operando* RH 100% conditions at (e) 650 and (f) 300 mV (the anode is at the top, the cathode is at the bottom) and Pt bump intensity in gray is overlaid to locate the membrane position with respect to the CLs.

First, the *d*-spacing of the ionomer peak is analyzed under reference conditions with an inert nitrogen feed (see Figure 9a and b for representative SAXS profiles of the membrane). The *d*-spacing is found to grow with increasing RH as previously reported in the literature^19,26,41^ (see Figures 9, S4, S5, and S9). Martens *et al.*(26) reported similar *d*-spacing values determined by SAXS-CT, though the exact equivalent weight of the used membrane is not reported. The actual *d*-spacing observed in the *operando* cell is, however, smaller (about 1 nm) than the values reported in the literature for Nafion XL.^19^ The X-ray beam probes the membrane both in the active cell domain and under the gasket area, which could likely result in an underestimation of the *d*-spacing in the active cell area.

During cell operation, two levels of relative humidity (RH 50% and RH 100%), both at two different voltages (650 and 300 mV) were investigated. For each of the two voltages, different locations of the cell were probed. At 650 mV and RH 50%, yielding a current density of about 0.13 A/cm^2^, the membrane hydration increases only slightly compared to the dry reference case, whereas at RH 100% the *d*-spacing increases clearly from 3.3 to 3.6 nm because of the higher rate of water production at about 0.36 A/cm^2^ (see Figure 9a,c). The current densities at 300 mV are similar at 1.15 A/cm^2^ (RH 50%) and 0.99 A/cm^2^ (RH 100%). Hence, the change in *d*-spacing to the *ex situ* values is 0.3 nm at both RH levels (see Figure 9b,d).

A gradient of the *d*-spacing, which is increasing in the TP direction from the anode to the cathode, can be clearly seen during operation at RH 50% and RH 100% at 300 mV (see Figure 9b,d). At 650 mV, a gradient of the *d*-spacing is also seen during operation at RH 100% and its wet reference at RH 100% particularly near the membrane–cathode CL interface (Figure 9c,d). The remaining dry and wet references are observed to be relatively homogeneous for both locations. It is likely that the scattering of the Pt particles contributes as an artifact to the ionomer *d*-spacing analysis near the membrane–cathode CL interface, contributing also to nonhomogeneous hydration of the wet reference case at 650 mV.

Next to the TP gradient in membrane hydration, 2D maps of the local membrane *d*-spacing provide detailed insights into the difference of the two examined locations and their heterogeneities (see Figure 9e,f). While the 650 mV location is quite wavy at the membrane–CL interface, the 300 mV location is rather flat. This explains the difference in the width of the PTFE peak in the TP profiles (see the black dashed line in Figure 9c,d). The location where the 300 mV condition was probed was possibly more parallel to the beam compared to the 650 mV probing location. Overall, no anode dry out was found as reported for thicker PFSA membranes.^43^

### Misalignment of MEA Layers Due to Membrane Swelling

**Figure 10:**
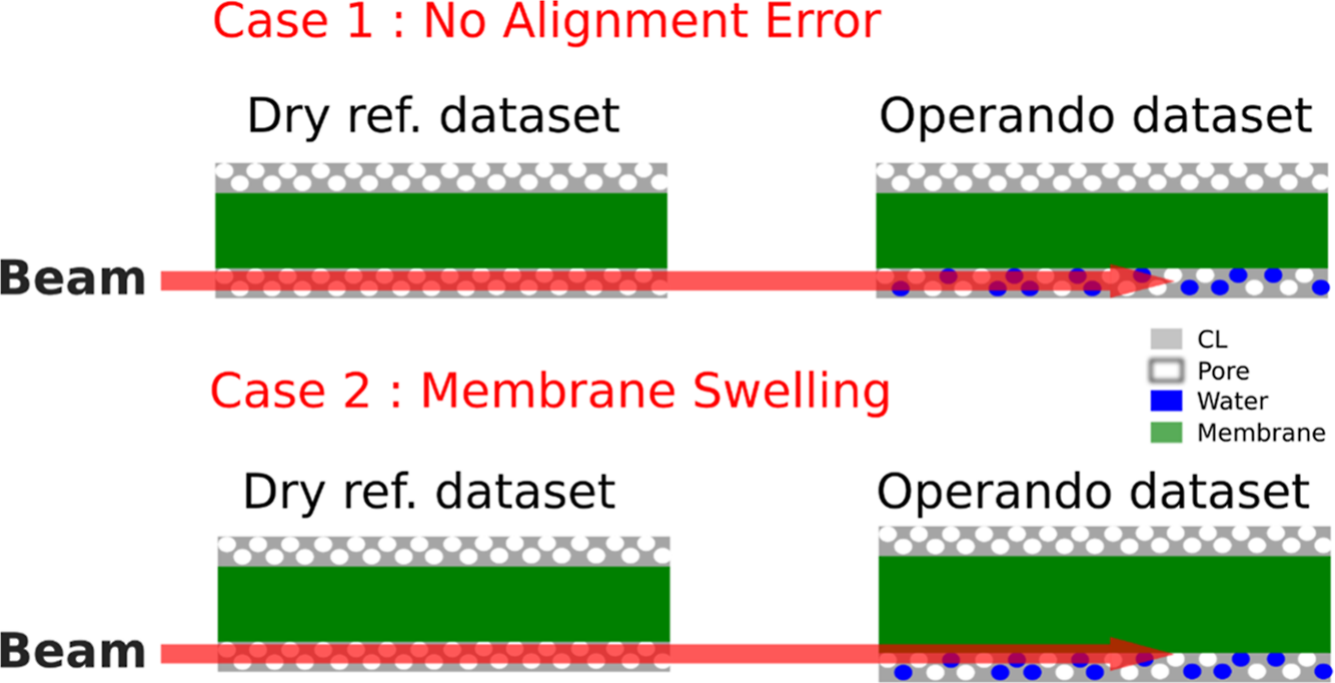
Schematic of the misalignment problem due to membrane swelling. The very same location of the CL (gray) or membrane (green) needs to be probed to determine the correct saturation level or hydration state, respectively.

As the membrane becomes more hydrated, the PFSA part of the membrane, composed of a PTFE-rich reinforcement layer sandwiched by two layers of PFSA, is expected to swell and thus the whole membrane. This can complicate the CL saturation level evaluation since the CL in the *operando* data set may not be at the same position as in the dry reference (schematically outlined in Figure 10). The correct saturation level can be detected well when the *operando* and the dry data set are well-aligned if there is no observable membrane swelling. In reality, the CL saturation is difficult to determine if the membrane swells. Instead of probing the water content in the CL only, contribution from both the CL and membrane being probed together may mislead the analysis.

For the used Nafion XL membranes, TP swelling due to water uptake is expected to be ≈ 6 μm for a single Nafion XL membrane,^19^ which might spread evenly to about ≈3 μm misalignment for anode and cathode CLs, each. With a step size of 5 μm for the low-loading MEA, the predicted swelling at the anode and cathode is estimated to be less than 1 pixel. Independent of membrane swelling, a mixture of the membrane and CL may be in the beam path that cannot be avoided at such a scanning step size.

Hence, the following misalignment analysis is aimed at isolating the bias of potential misalignment to the CL saturation level. It is limited to the maximum misalignment of 1 pixel. The idea here is to compare the dry reference SAXS data of neighboring pixels as a worst-case scenario of misalignment, which should be free of any influence by saturation changes as compared to the operating conditions. By doing so, a threshold is derived, which serves to decide whether the detected amounts of water could be a result of misalignment or stem from actual water in the CL pores.

As a first step, we assume that the neighboring pixel toward the membrane at the membrane–CL interface (*i* – 1) would be shifted to the identified location of the CL (*i*) by membrane swelling. If the difference in the scattering intensity from such an assumed misalignment is larger than the saturation level of the liquid water detected in the *operando* data, then the saturation level is regarded as unreliable. Moreover, an increase in the PTFE peak (≈12.8 nm^–1^) at the CL location between the dry reference and the *operando* data may also indicate a misalignment, where CL scattering is overlaid with more PTFE scattering contributions from the membrane. Such cases are also excluded. Together this translates into the two criteria below that must be fulfilled to provide a reliable measure of the cathode CL saturation level
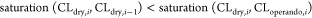
1
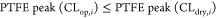
2

**Figure 11:**
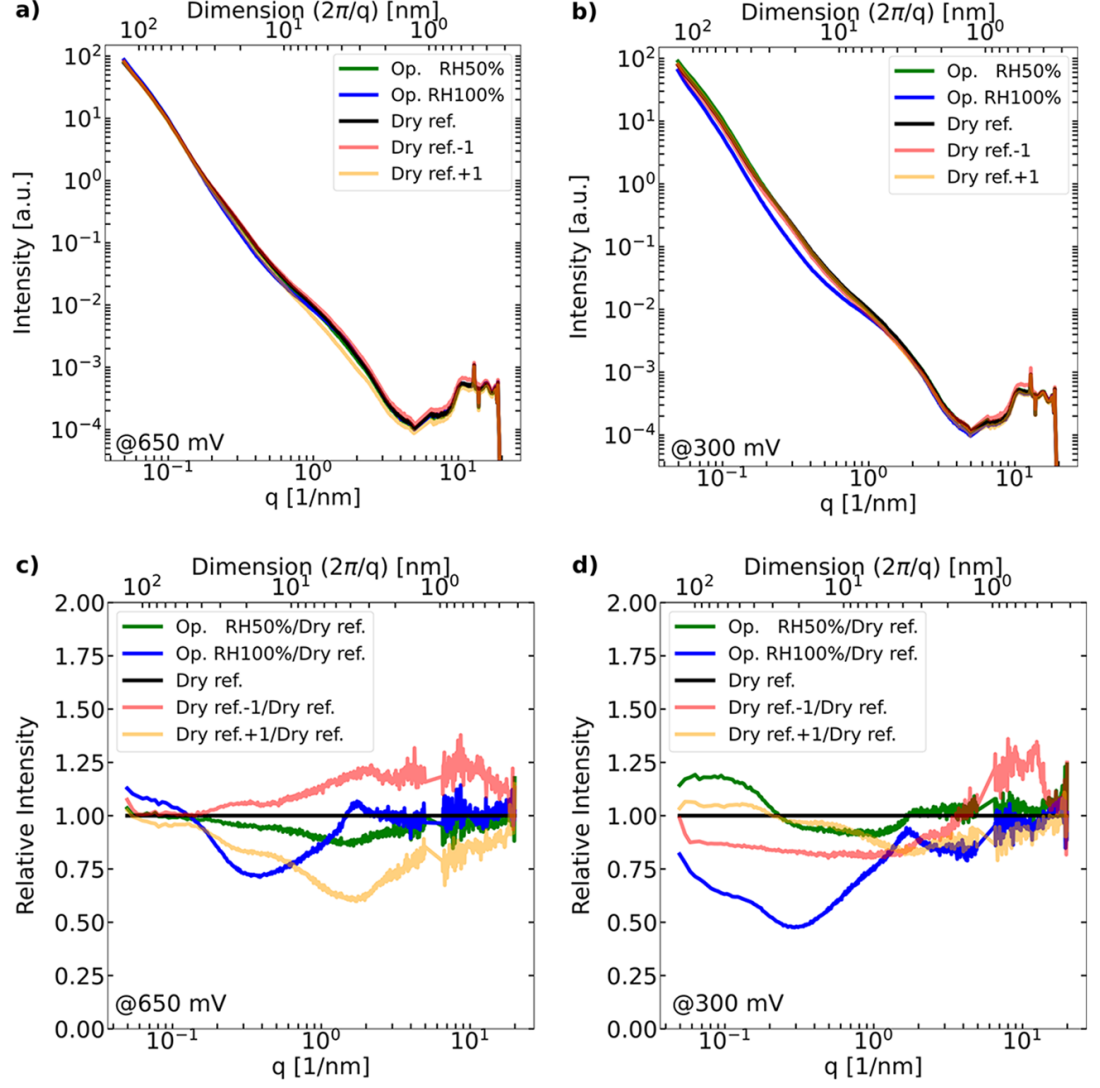
Scattering intensity (a,b) and relative intensity (c,d) versus *q* profiles for the dry reference and *operando* conditions together with the misalignment tests comparing ±1 neighboring TP scan position to the dry reference for the two voltage conditions and their corresponding scanning locations of (a,c) 650 and (b,d) 300 mV.

SAXS intensity profiles for operando RH 50%, RH 100% at both 650 and 300 mV (see Figure 11a,b) were normalized with dry references at RH 50% to better visualize the change in intensity relative to dry SAXS profiles (see Figure 11c,d). In order to see if the two criteria above were satisfied, the neighboring pixels of dry references were examined (dry ref −1 and dry ref +1). The relative intensities of dry ref −1 normalized by dry ref were higher than one at 650 mV (see Figure 11c). Hence, the profiles at both RH 50% and RH 100% are compatible with the criteria of higher saturation level than the alignment bias. Also, the PTFE peak does not increase at 650 mV for both RH 50% and RH 100%. Hence, the two *operando* profiles are regarded as reliable. The neighboring scan toward the MPL (dry ref +1) reveals a missing Pt bump, signaling that the neighboring TP scan position was already the MPL. In future studies, the WAXS signal of Pt at *q* ≈ 30 nm^–1^, which was beyond the WAXS detection range in the current study, could be used to give additional confidence in the location of the CLs.

At 300 mV voltage (Figure 11d), the TP scan position toward the membrane (dry ref −1) exhibits more intensity decrease than the *operando* profile at RH 50% but less than the one of RH 100%. Hence, the SAXS profiles at RH 50% have to be rejected to be analyzed further, while the profile at RH 100% is considered reliable. The TP scan position toward the MPL (dry ref +1) exhibits a lower Pt bump. Therefore, there is still the CL in the beam path at the probing location of dry ref +1. As a consequence, only *operando* cathode CL data at RH 100% (both at 650 and 300 mV) will be further analyzed in this work.

In follow-up studies, a smaller step size without inflicting higher radiation dose to avoid damage and/or a smaller beam size are needed to more finely resolve the CL and to identify its bulk location. Such higher resolution would also allow for precise registration of the membrane location between dry and *operando* SAXS data.

### Operando CL Saturation

**Figure 12:**
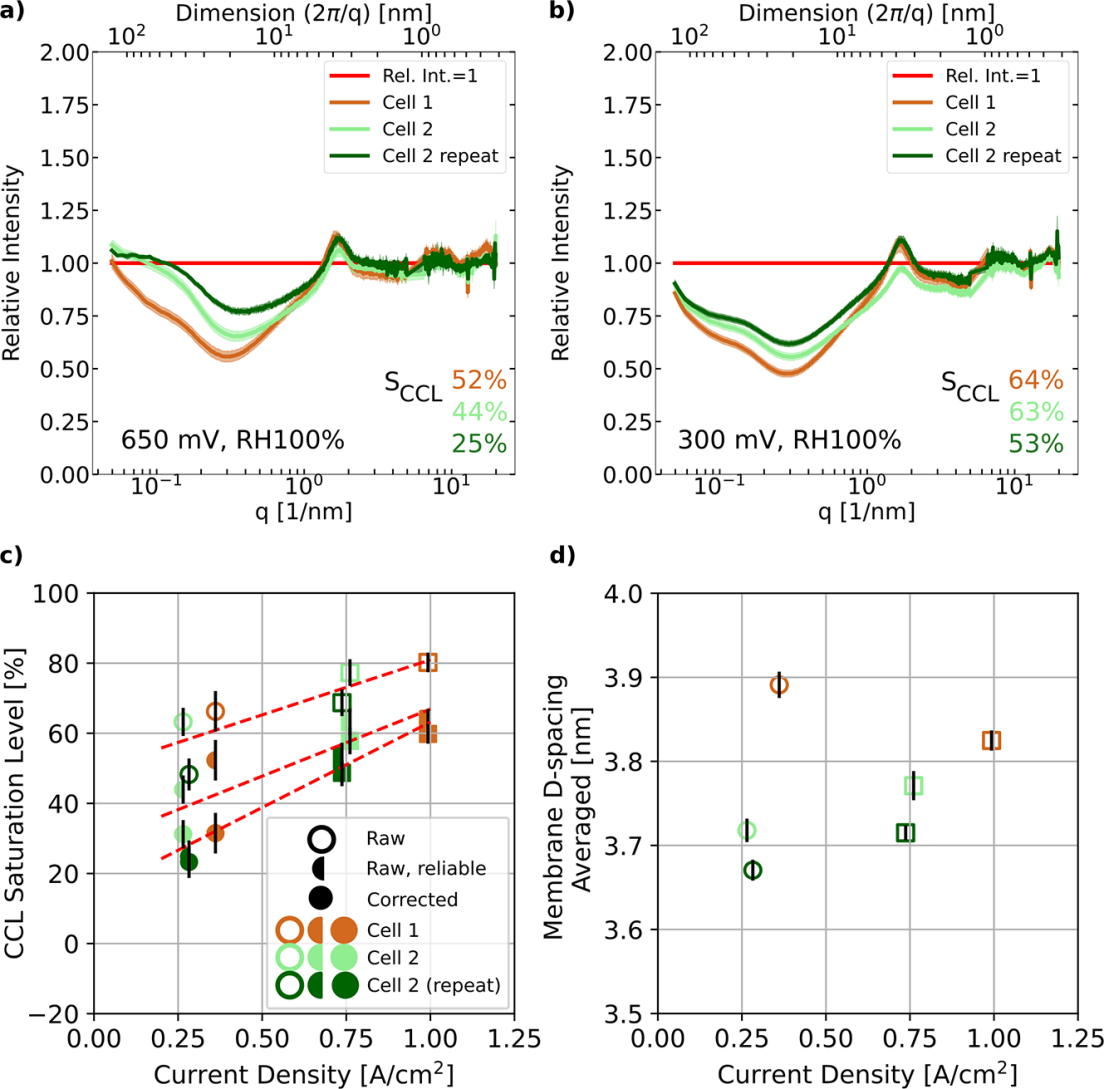
Cathode CL relative intensities (normalized to dry reference acquired at the same measurement spots) with reliable raw saturation level values at (a) 650 and (b) 300 mV with the standard error of the mean shown by transparent colors calculated for the different cell locations. Cathode CL saturation levels (c) and membrane hydration level (d) averaged over the scanned area at operating conditions of RH 100% at 50 °C. Raw cathode CL saturation levels without considering misalignment criteria are shown as unfilled symbols, the raw but reliable spots (considering eqs 1 and 2) as half-filled symbols, the reliable and corrected ones (considering eqs 1–3) as filled symbols, with 650 mV as circles and 300 mV as squares. Trend lines in dashed orange are provided as guides to the eye.

Before the CL saturation during cell operation is addressed, the reproducibility of the SAXS measurements in the new operando cell is discussed. Azimuthally integrated scattering intensities normalized to the dry reference scattering data of the cathode CL are shown for two different cells in Figure 12. Each relative intensity curve (see Figure 12a,b) aggregates data from multiple line scans taken in close vicinity (all within ≈360 μm in-plane direction) at steady state conditions that falls into the two reliability criteria stated above (see eqs 1 and 2). Only the highest saturation level in each vertical line scan was regarded as the averaged relative intensities. The error bars are small for the whole *q*-range but increase slightly with increasing scattering angle, which is to be expected given the fact that the signal-to-background noise ratio of the pure scattering signal is also lower in this *q*-range. The relative SAXS profiles are quite different for the two cells, with up to ≈30% difference, in particular at high cell voltage and at *q* values below 1 nm^–1^. These changes in the SAXS profiles are caused by differences in water saturation in pores of about ≈6 nm and larger. The difference to a repeated measurement acquired at a different location (>5 mm apart) in cell 2 is smaller but still up to ≈15%. Also here, the relative SAXS intensities are more similar at the 300 mV low voltage condition. These deviations between the two cells can have different reasons. It may be a result of the remounting of the MEAs into the *operando* cell, which causes not well reproducible current densities at the same cell voltage. Also, the inhomogeneity of the CL and specifically the MPL with the cracks of the used SGL29 BC GDL, could influence the local saturation in the CL and thereby the local SAXS signal. Since the scanned 650 and 300 mV locations are different, it could be that just by chance all 300 mV spots had a more similar CL or MPL structure compared to the 650 mV spots.

The cathode CL saturation levels (CCLs) as shown in Figure 12c were extracted from the scattering intensity profiles by invariant calculation.^39^ The saturation levels were derived from 10 nearby line scans in order to provide statistical evidence with the standard error of the mean not exceeding ±5%. Only the highest saturation levels per vertical line scan are incorporated to the averaged saturation levels, if more than one point of a line scan was identified as the CCL. The raw saturation numbers without regard of the alignment criteria increase from 50 to 75% for current densities around 0.3 A/cm^2^ to 70 to 80% at current densities of 0.75 to 1 A/cm^2^, respectively. Applying the two reliability criteria of eqs 1 and 2 to the raw saturation values reduces the average CCL saturation by almost 20% for all current densities. Again, the cell-to-cell variation is higher than the in-cell fluctuations and is more pronounced at lower current densities.

However, the cathode CL saturation level may be still biased by an intensity decrease in the SAXS data caused by a misalignment of the CL in the dry and wet states due to membrane swelling even after considering only intensity changes that are larger than the potential bias. To correct for this, the potential saturation error due to misalignment is estimated by interpreting the scattering intensity differences between the dry reference SAXS data of the CL location *i* and the neighboring line scan location toward the membrane *i* – 1 using the invariant calculation as biased water saturation [saturation_bias_(CL_dry*,i*_*,*CL_dry*,i*–1_)] and subtracting it from the raw saturation level of location *i*
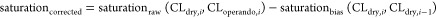
3

The deviation of the corrected saturation levels from the reliable raw saturation levels (filled symbols in Figure 12c) is found to be stronger for lower current densities, where CL saturation decreased by 10% to about 30%, whereas saturation levels at higher current densities decrease only by 5 to 50 to 60%. Excluding nonreliable CCL locations from the saturation analysis is found to have a stronger influence on the saturation values than the correction by potential misalignment. Overall, the trend of saturation, increasing with increasing current density, is more pronounced in the corrected data, with significantly less scatter at low current densities. The corrected saturation values are pointing toward a linear relationship between CL saturation and current density for the investigated operating conditions whereas the raw saturation values could be only reproduced by a nonlinear relationship, in particular for current densities below 0.5 A/cm^2^. Detailed 2D maps of the spatial distribution of the raw saturation, its bias, and the corrected saturation can be seen in Figures S10–S12, respectively.

In our earlier work,^39^ we outlined how representative 3D pore filling simulations could be used to interpret the scattering data in a sense of pore size specific filling. Even though in this work no specific model fit is applied, it is possible to draw qualitative conclusions on the characteristic length scales of water accumulation and the corresponding pore sizes filled. The strongest decrease in the scattering intensity (Figure 12) is observed for *q*-ranges below 1 nm^–1^ (corresponding to pores of 6 nm or larger). Only with higher saturation values, the decrease in scattering expands further toward smaller *q* or larger pores. Such a trend suggests that small pores are filled already at lower current densities while larger pores fill only at higher saturation levels but even then not completely (*i.e.*, not all pores of larger size were filled). If all pores were filled with liquid water, the relative scattering intensity would show a constant plateau of lower scattering intensity at small *q*-values.

The maximum decrease in scattering is observed at *q* values of about 0.3–0.4 nm^–1^ or about 20 nm pore dimensions. Pores up to this diameter are probably filled almost completely while larger pores remain only partially filled, although not completely empty. If no pores of about 100 nm would contain liquid water, an increase of the low *q* SAXS intensity should have been observed.^39^ Since this is not the case for the reliable measurements, we conclude that at 100% RH already at 0.3 A/cm^2^ the larger pores contain already some water, resulting in the small change of scattering intensity at low-*q* (<10^–1^ nm^–1^). At higher current densities and higher saturation levels, a larger fraction of the amount of water in the larger pores increased further since the scattering intensity at *q* < 0.3 nm^–1^ decreases. At about 1.5 nm^–1^, an intensity maximum (or a peak) can be found for all 100% RH conditions, which is most likely due to the water uptake in the ionomer. The decreased scattering intensities between 2 and 6 nm^–1^ can be assigned to liquid water in the primary pores of the Ketjenblack carbon support of the catalyst. The saturation of these micropores increases with current density and seems to plateau at about 0.75 A/cm^2^ and higher current densities.

CLs are well-known to have different local wettability inside the structure.^44^ Pt nanoparticles are hydrophilic, carbon supports are hydrophobic, the wettability of the two components, however, can change depending on the ionomer coverage.^44^ In our data, the intensity decrease at smaller real space dimension may suggest capillary condensation that frequently happens first in smaller pores in the case of hydrophilic substrate, *i.e.*, Pt-filled mesopores of the carbon,^45^ in combination with water generated in the vicinity of the Pt particles that reside on the carbon surface (larger pores) and inside the carbon support (smaller pores). Given the hydrophobicity of the carbon support, small capillary pressure in the larger pores may induce the filling of larger pores at higher current densities.^45^

The averaged membrane *d*-spacing dependency on the current density is summarized in Figure 12d. The *d*-spacing seems to increase with current density, besides a potential outlier at 650 mV for cell 1. The in-cell repeat for cell 2 reproduces consistently the trend in cell 2. The observed *d*-spacing values are lower compared to 4.5 nm reported at room temperature under RH 98% vapor-saturated *in situ* conditions in.^19^ The reason for the deviation remains unclear but could stem from differences in the temperature or membrane orientation during the SAXS measurements. As already mentioned in the Membrane Hydration section, Martens *et al.*(26) showed similar low *d*-spacing values in operando conditions using SAXS tomography for a 20 μm thin reinforced membrane. Using literature data of a Nafion 117, though not reinforced, membrane,^46^ the *d*-spacing values in Figure 12d would translate to lambda values of 8.4–9.4 for *d*-spacing of 3.7–3.8 nm, respectively, which appear rather low for 100% RH conditions and liquid water detected in the nearby CL. For accurate lambda translation from *d*-spacing values, additional membrane specific calibration efforts within the operando SAXS cell will be needed in the future as the conversion is specific to the type of membrane.

## Conclusion

A scanning SAXS-compatible *operando* PEFC has been developed with powerful capabilities to identify the different components of PEFCs and to diagnose water levels, especially in the CLs and the membrane. The cell design and material selection, aimed at X-ray transparency and suppressing parasitic scattering coming from the setup, resulted in a unique operando SAXS cell design with just a 1 mm material thickness of the active cell components in the beam path. Several challenges that had to be overcome to achieve reliable insights were thoroughly analyzed and described, and mitigation approaches were provided.

*Operando* scanning SAXS at 50 °C cell temperature was employed and allowed to differentiate between the 10 μm thick anode and cathode CLs and the ≈30 μm thick membrane in between with a TP scanning step size of ≈5 μm. A gradient in membrane hydration at high current densities (300 mV) from the anode to cathode has been observed based on a 0.1 to 0.2 nm difference in nanodomain spacing. After accounting for potential misalignment due to membrane swelling, we could show that the cathode CL saturation increases with current density, from about 30% at 0.3 A/cm^2^ to about 60% at 1 A/cm^2^. An inspection of the *q*-range in which the scattering intensity decreases due to liquid water accumulation in the CL indicates a preferential filling of smaller pores and an incomplete filling of larger pores.

For future work, finer SAXS scanning step size at similar or lower X-ray doses could reduce the misalignment due to membrane swelling and the related saturation uncertainty. Combined with representative pore filling simulations the full potential of *operando* SAXS saturation analysis could be unfolded. The synergistic development of the *operando* setup and the appropriate data analysis tools will enable detailed mechanistic investigations and an improved design of CL and MPL in PEFC and other electrochemical devices.

## Supporting Information Available

Schematic of the experimental setup details, catalyst layer SEM images, 2D maps of the d-spacing distribution, electrochemistry time series data for cell 1 and cell 2, polarization curves, and water distribution in the anode and cathode CLs (PDF)
