# Supplementary material for: Operando Scanning Small-/Wide-Angle X-ray Scattering for Polymer Electrolyte Fuel Cells: Investigation of Catalyst Layer Saturation and Membrane Hydration– Capabilities and Challenges
Source: ACS Appl Mater Interfaces. 2024 May 13;16(20):25938–52. doi: 10.1021/acsami.3c11173 (PMC11129111; doi:10.1021/acsami.3c11173)
Supplement: Supplementary file 1 — am3c11173_si_001.pdf [file am3c11173_si_001.pdf]

Supporting Information

Operando Scanning Small/Wide Angle *X*-ray Scattering for Polymer Electrolyte Fuel Cells: Investigation of Catalyst Layer Saturation and Membrane Hydration– Capabilities and Challenges

Kinanti Aliyah[1], Christian Appel[2], Timon Lazaridis[4], Christian Prehal[5], Martin Ammann[1], Linfeng Xu[1], Manuel Guizar-Sicairos[2,3], Lorenz Gubler[1], Felix N. Büchi[1], Jens Eller[1]*

Affiliations

[1]**Electrochemistry Laboratory, Paul Scherrer Institut, CH-5232 Villigen PSI, Switzerland**

[2]**Photon Science Division, Swiss Light Source, Paul Scherrer Institut, CH-5232 Villigen, Switzerland**

[3]**Institute of Physics, École Polytechnique Fédérale de Lausanne (EPFL), CH-1015 Lausanne, Switzerland**

[4]**Technical University of Munich, Chair of Technical Electrochemistry, Department of Chemistry and Catalysis Research Center, D-85748 Garching, Germany**

[5]**Department of Information Technology and Electrical Engineering, ETH Zurich, CH-8092 Zurich, Switzerland**

*corresponding author

Keywords: polymer electrolyte fuel cell, water management, catalyst layer, membrane hydration, operando small-angle X-ray scattering

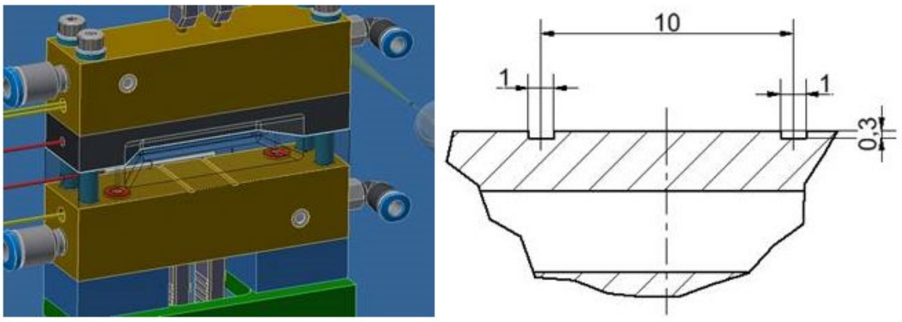

Figure S1 Rectangular slits on the interface of the current collector and flow field with defined width and height. The alignment of the cell with respect to the beam is monitored by the transmission from the interface to the interface of the slits in both horizontal and vertical directions.

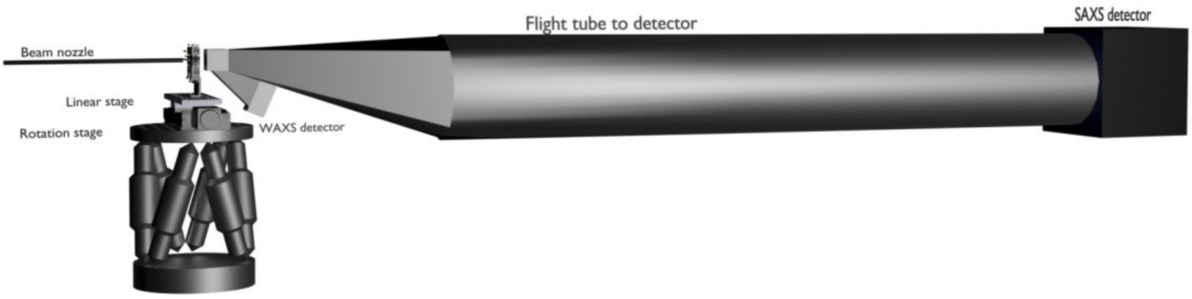

Figure S2 Schematic of the experimental setup to ensure precise alignment. Incorporation of hexapod/rotation state and linear stage.

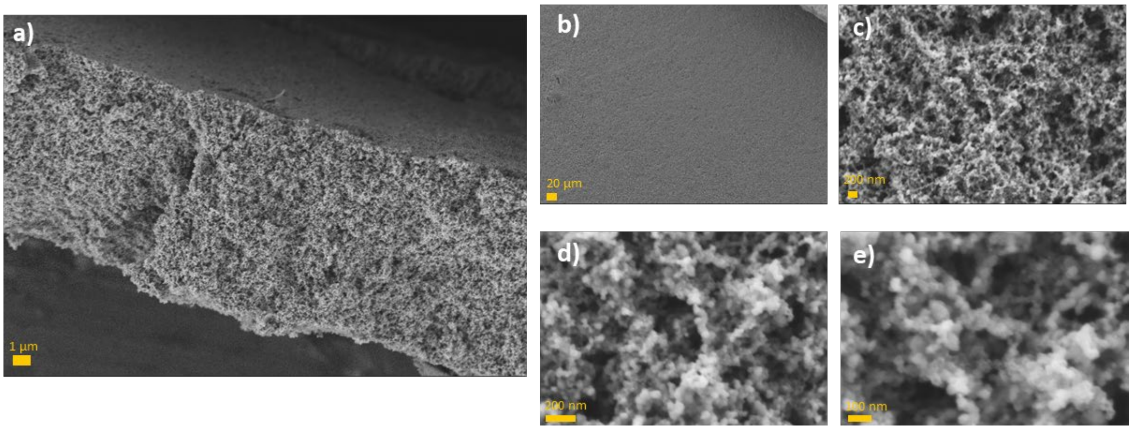

Figure S3 Scanning electron microscopy images of catalyst layer on PTFE decal used in the study at various magnifications, a) cross-section, b-e) surface images.

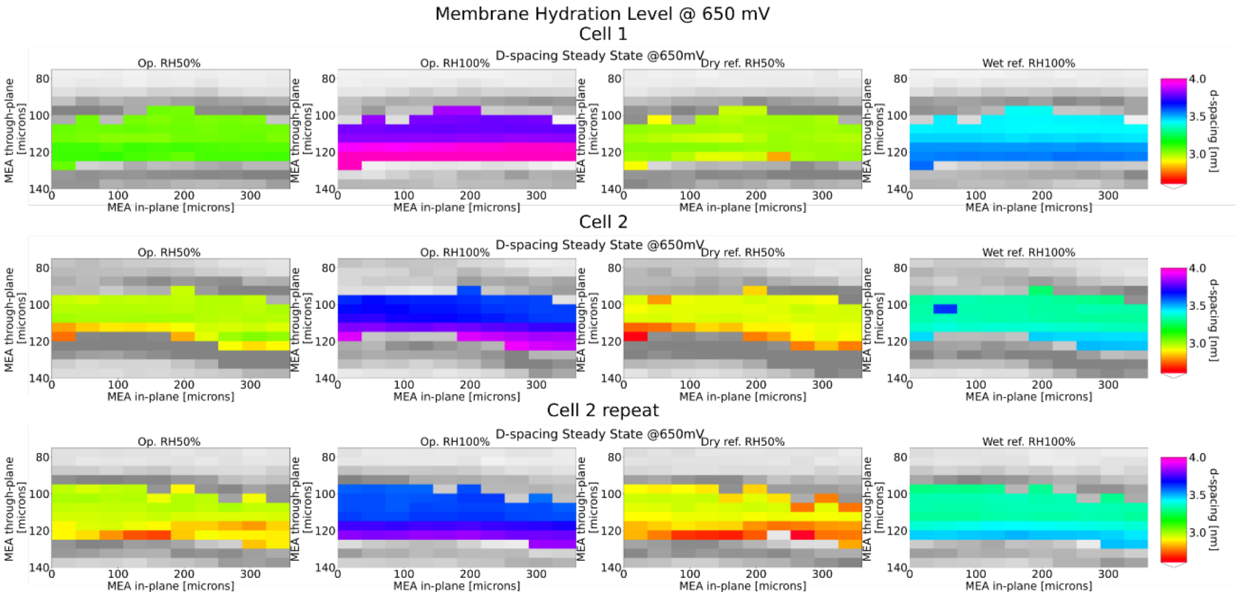

Figure S4 2D map of the d-spacing distribution of the scanned area in Cell 1 (top row), Cell 2 (middle row), and Cell 2 repeat (bottom row), at operando condition 650 mV, dry and wet references. Pt bump intensity is overlaid on the d-spacing map in grey to help locate the membrane.

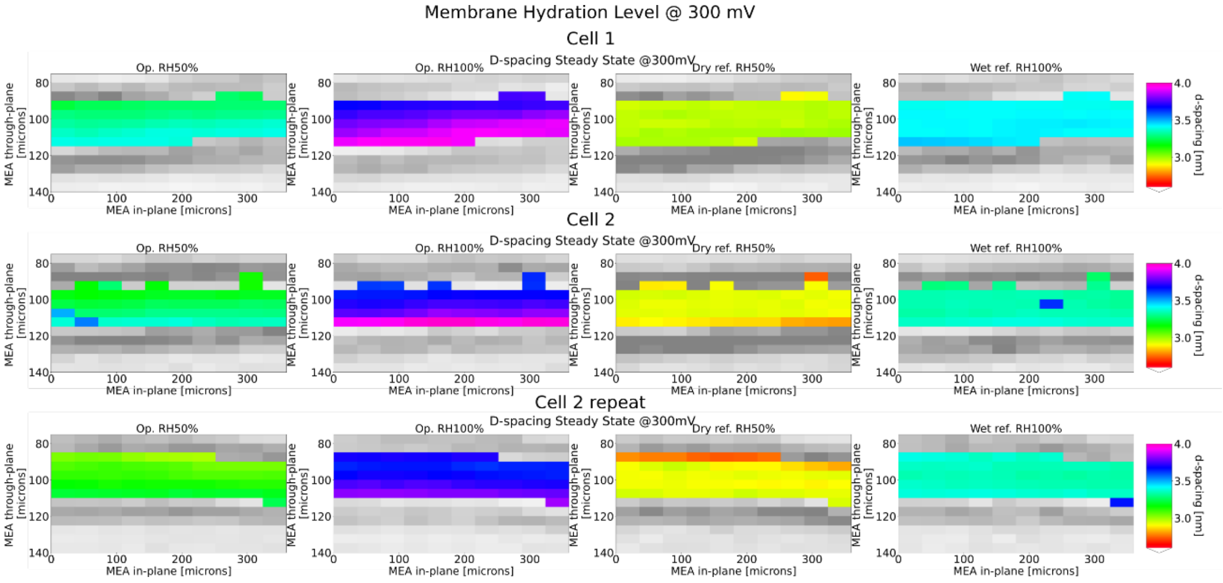

Figure S5 2D map of the d-spacing distribution of the scanned area in Cell 1 (top row), Cell 2 (middle row), and Cell 2 repeat (bottom row), at operando condition 300 mV, dry and wet references. Pt bump intensity is overlaid on the d-spacing map in grey to help locate the membrane.

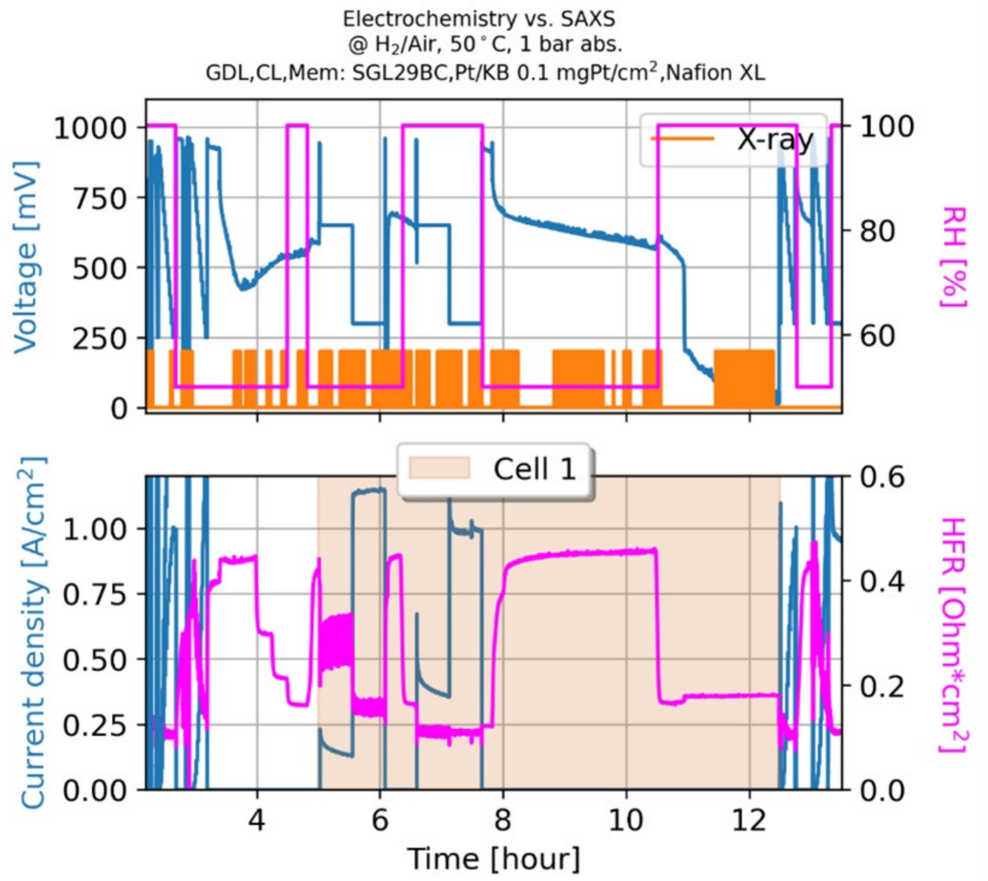

Figure S6 Electrochemistry time series data for Cell 1. SAXS was measured at RH50% and RH100% at 650 mV and 300 mV, followed by the drying step, acquiring dry references at RH50% and wet references at RH100%. Polarization curves were taken after reference measurement to compare with polarization curves taken before measurement.

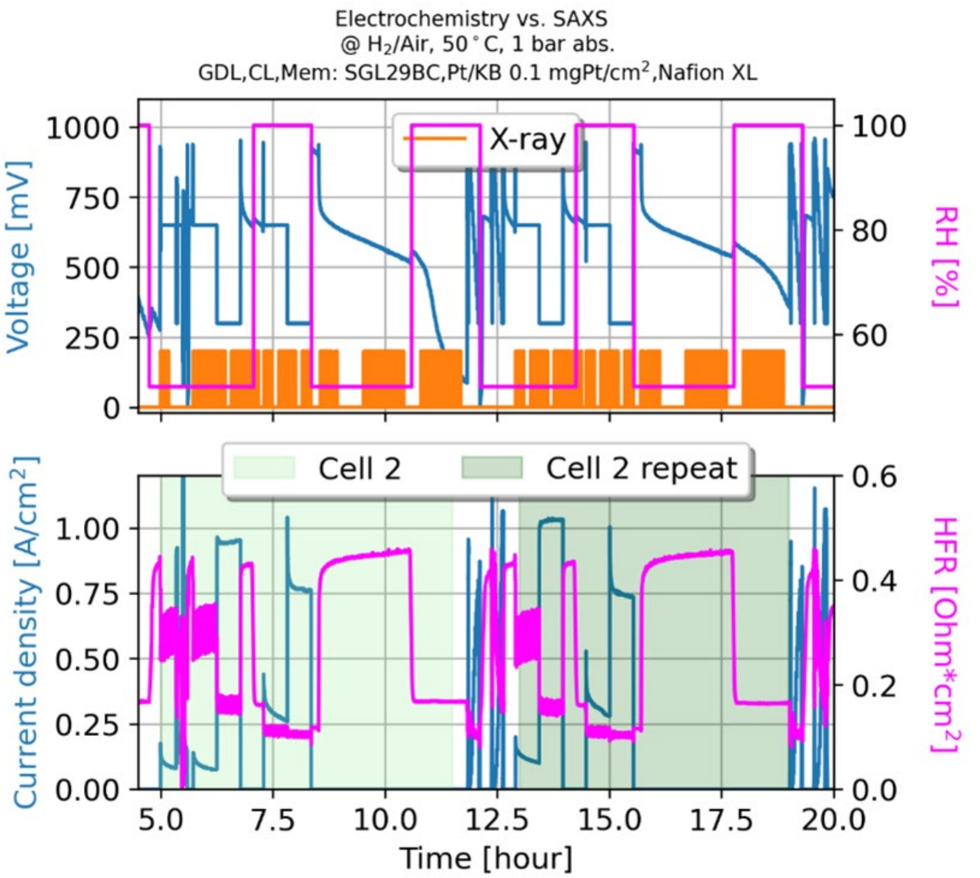

Figure S7 Electrochemistry time series data for Cell 2. SAXS was measured at RH50% and RH100% at 650 mV and 300 mV, followed by the drying step, acquiring dry references at RH50% and wet references at RH100%. Polarization curves were taken after reference measurements to compare with polarization curves taken before the whole measurements. For this cell, an in-cell repeat or a repeat of exact measurements with the same MEA was carried out at different cell locations.

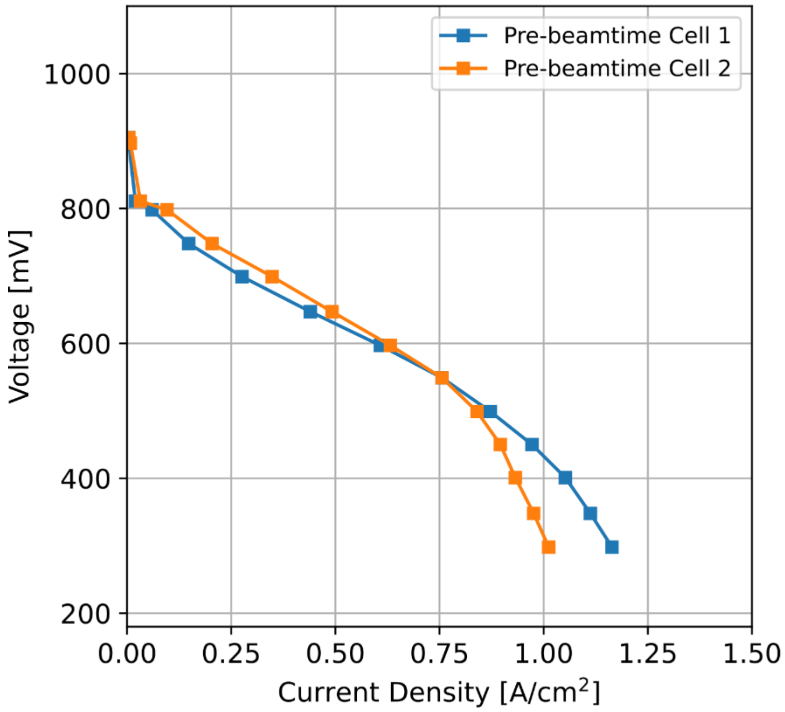

Figure S8 Polarization curves after preconditioning of cell 1 and 2 before disassembling and reassembling at the beamline. Operating conditions are at RH 100%, 50ºC.

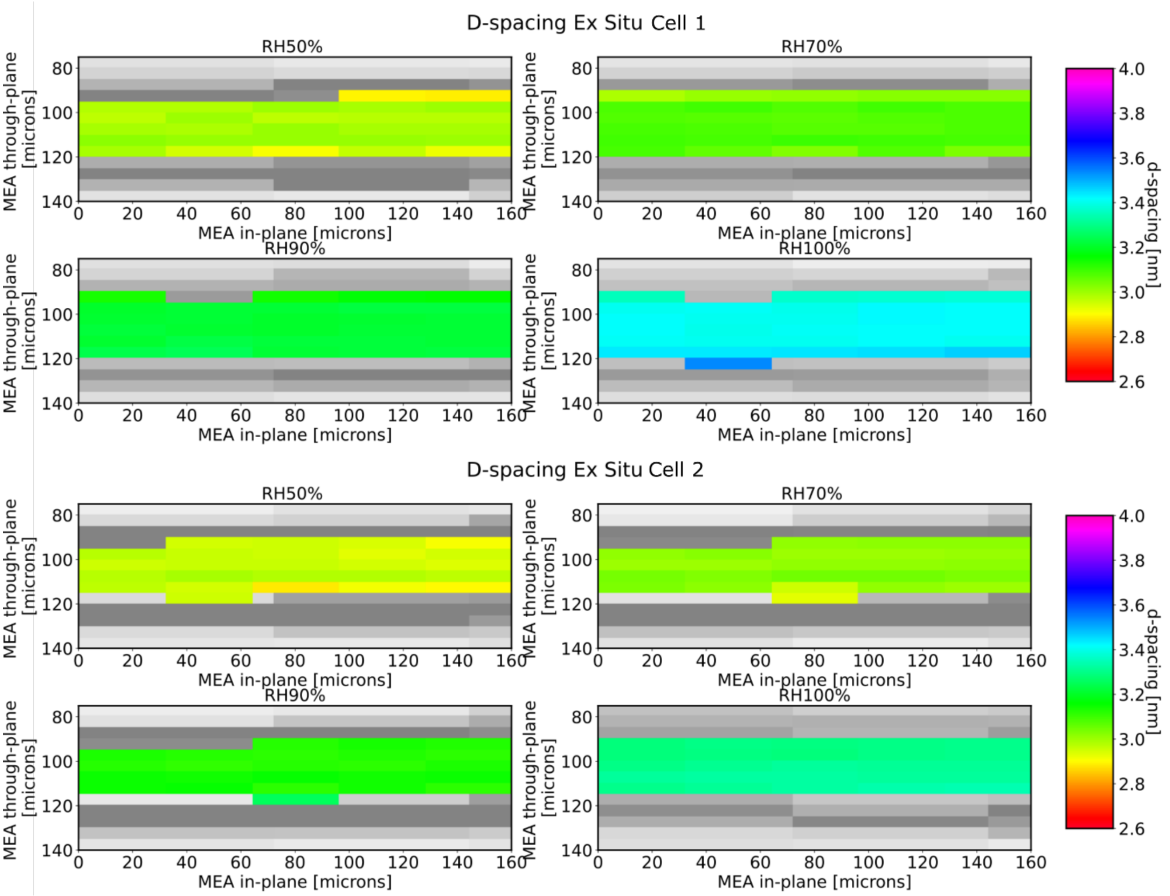

Figure S9 2D map of the d-spacing distribution of the scanned area in Cell 1 (top) and Cell 2 (bottom) at various relative humidity (RH 50%, 70%, 90%, 100%). Pt bump intensity is overlaid on the d-spacing map in grey to help locate the membrane.

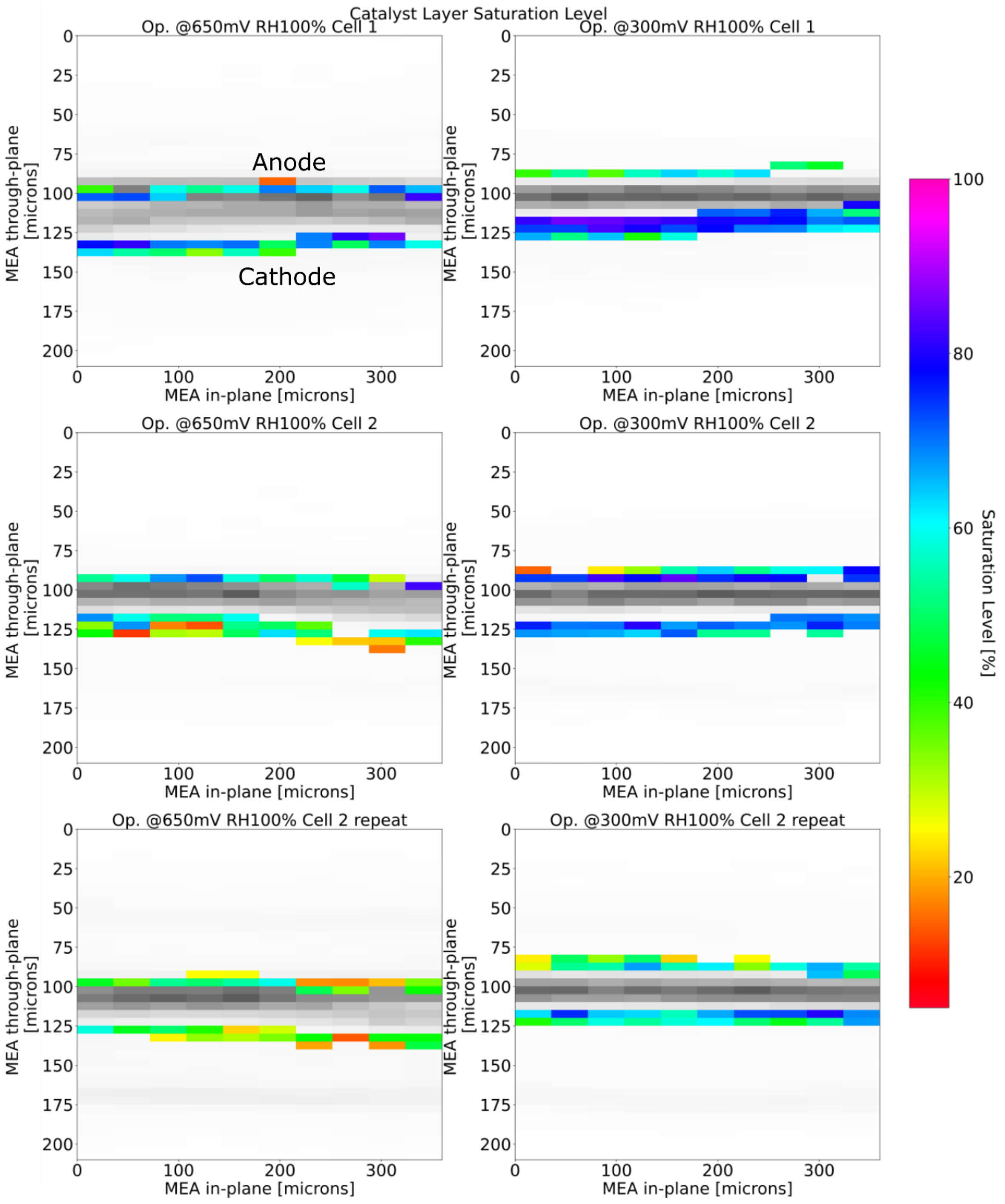

Figure S10 Water distribution in the anode and the cathode CL without applying the misalignment criteria. PTFE peak intensity is overlaid on the CL saturation map in grey to help locate the membrane.

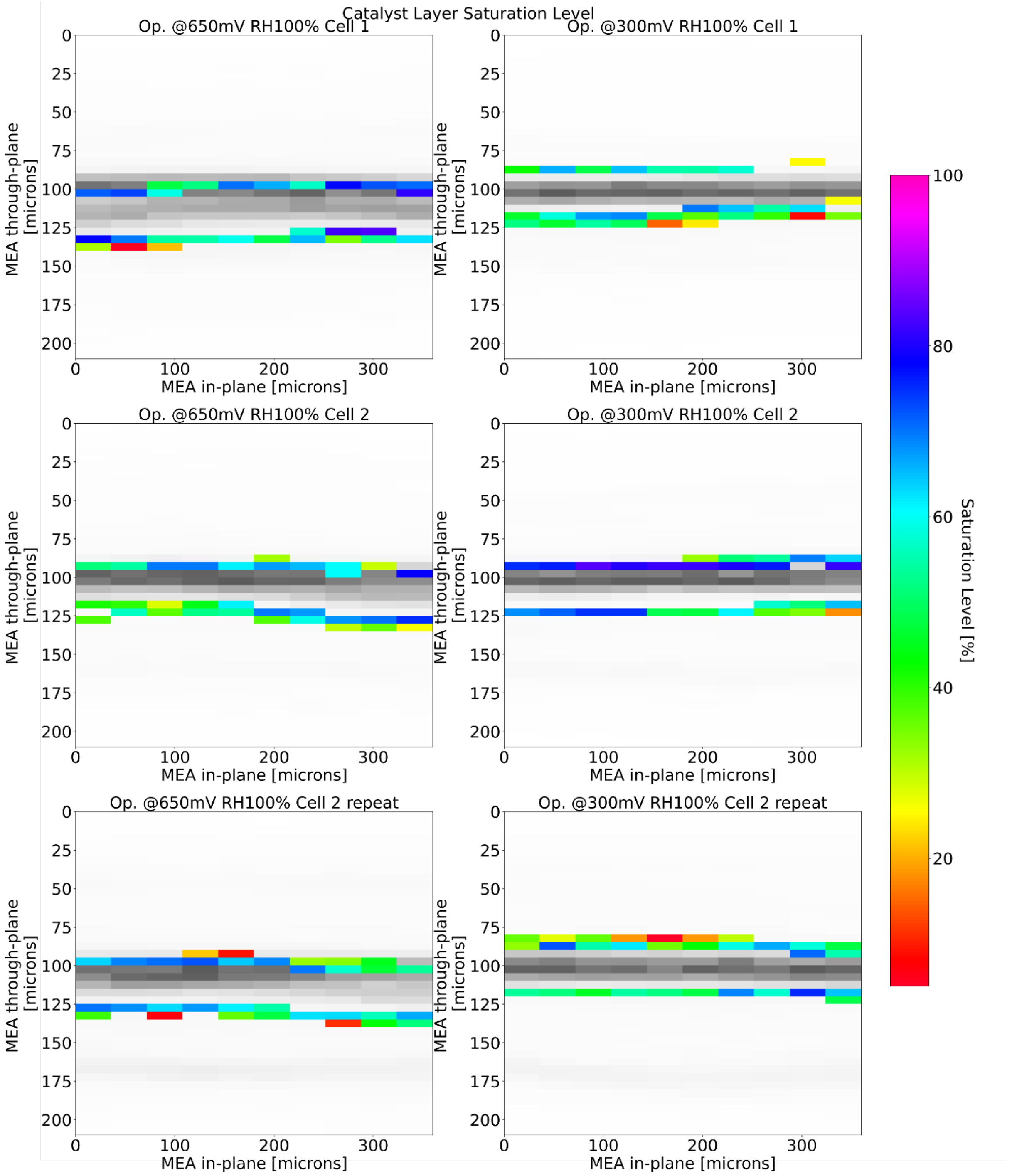

Figure S11 "Water" distribution in the anode and the cathode CL simulated from misalignment criteria. Dry-1 is as "wet" at the cathode, and dry+1 is as "wet" at the anode. PTFE peak intensity is overlaid on the CL saturation map in grey to help locate the membrane.

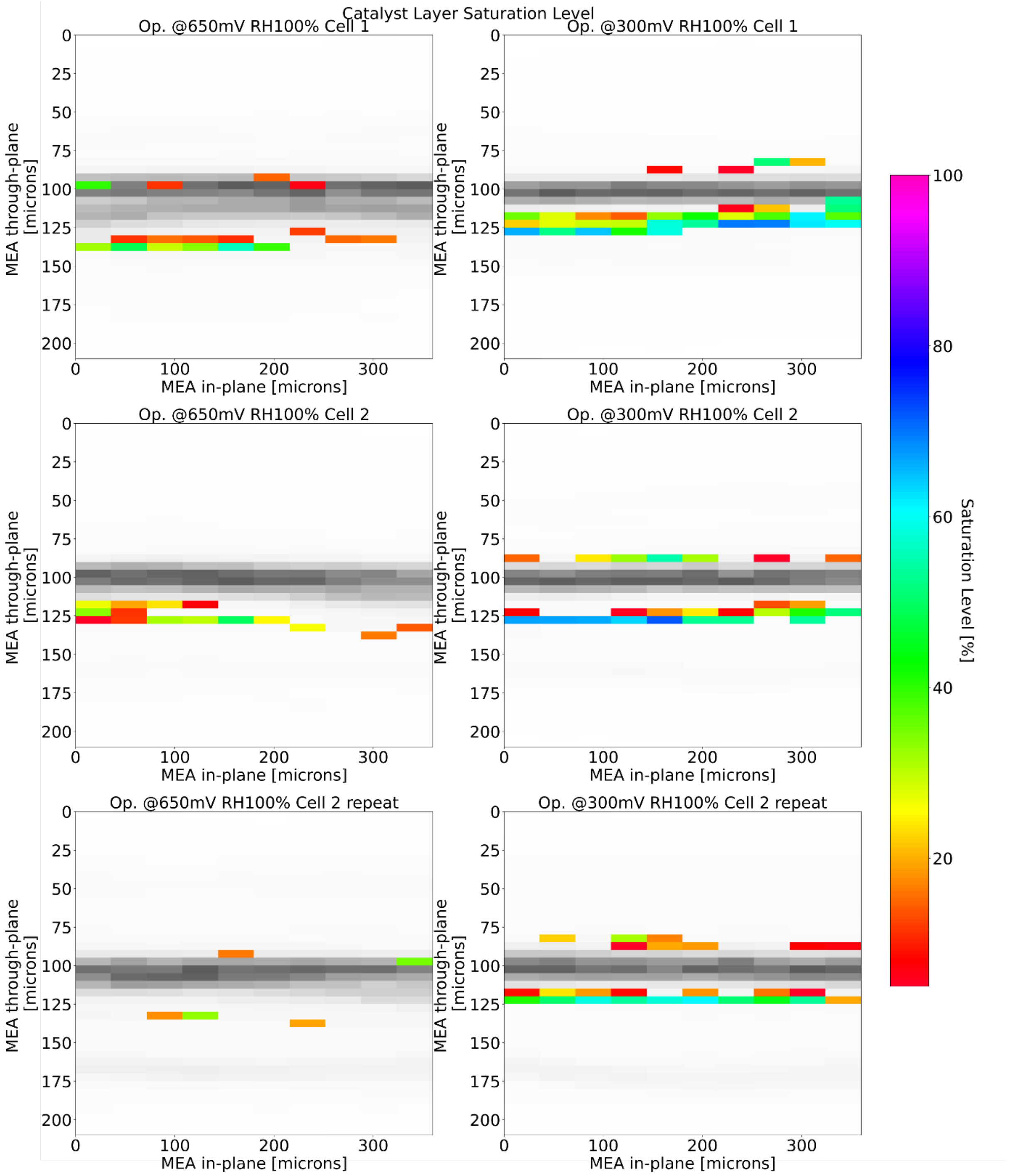

Figure S12 Water distribution in the anode and the cathode CL corrected by subtracting the misalignment saturation value. Water content in the anode CL is mostly smaller than the criteria. Hence, almost none of the anode CL saturation level remains. PTFE peak intensity is overlaid on the CL saturation map in grey to help locate the membrane.
